# Computational and experimental approaches to explore defense related enzymes conferring resistance in Fusarium infected chilli plants by regulating plant metabolism through nutritional products

**DOI:** 10.1371/journal.pone.0309738

**Published:** 2025-01-14

**Authors:** Muhammad Usman, Muhammad Atiq, Nasir Ahmed Rajput, Muhammad Sagheer, Ye Xia

**Affiliations:** 1 Department of Plant Pathology, University of Agriculture Faisalabad, Faisalabad, Pakistan; 2 Department of Plant Pathology, The Ohio State University, Columbus, Ohio, United States of America; 3 Department of Entomology, University of Agriculture Faisalabad, Faisalabad, Pakistan; University of Agriculture Faisalabad, PAKISTAN

## Abstract

Nutritional status being the first line of defense for host plants, determines their susceptibility or resistance against invading pathogens. In recent years, the applications of plant nutrient related products have been documented as one of the best performers and considered as alternatives or/and supplements in plant disease management compared to traditional chemicals. However, knowledge about application of plant nutrient related products for the management of destructive fungal pathogen *Fusarium oxysporum* f.sp. *capsici* and their impact on the components of the antioxidant defense system, especially in chilli plants, still needs to be discovered. Therefore, in this current study, we aimed to evaluate two nutrient fertilizers viz. Krystafeed and Micro Plus at three different concentrations by soil drenching method for their effects against the Fusarium wilt of chilli and investigate the components of the antioxidant defense system of chilli plants. Correlation and computational analysis on the components of antioxidant defense system in various pathways were performed to predict the suitable binding sites of mineral ions. Results indicated that the combination of Krystafeed and Micro Plus was found the most effective with (27.01, 26.59%) disease incidence, followed by Micro Plus (29.56, 32.35%) and Krystafeed (38.21, 41.15%), both in greenhouse and field conditions, respectively. Moreover, the combination of Krystafeed and Micro Plus significantly increased the concentration of SOD (27.53, 108.96)%, POD (37.29, 45.65)%, CAT (19.33, 95.33)%, H_2_O_2_ (22.13, 118.98)%, TPC (27.39, 17.37)%, chlorophyll *a* (21.80, 35.74)%, chlorophyll *b* (57.57, 18.25)%, total chlorophyll (30.21, 19.83)%, Tocopherol (13.08, 33.66)%, TrxR (5.03, 36.56)%, MDA (13.84, 54.79)%, ascorbate (4.72, 17.28)%, Proline (5.94, 59.31)%, and phytoalexin (Capsidiol) (11.33, 55.08)% in the treated plants of resistant and susceptible chilli varieties, respectively, as compared to the untreated plants. Pearson’s correlation heat-map analysis showed that all the enzymes of antioxidant defense system were found positively correlated with each other. It is concluded that the improvement of crop resistance by the application of plant nutrient related products may be viable alternatives to synthetic chemicals for managing Fusarium wilt disease of chilli and potentially other pathogens.

## 1. Introduction

Chilli pepper (*Capsicum annum*) is a key crop which fulfills the world’s spice and pharmaceutical needs. Its production is continuously hammering by different fungal, bacterial, and viral pathogens [1]. Among fungal diseases, Fusarium wilt is one of the most destructive diseases, which is caused by *Fusarium oxysporum* f. sp. *capsici* (*Foc*) [2, 3], leading to the reduction in yield from 68 to 71% of 32.3t/ha of world’s average green chilli production [2, 4], with a 44.94% disease incidence based on the previous study [5]. *Foc* is an asexual homothallic fungus [6], which remains persistent in soil for long time in the form of chlamydospores [7]. This pathogen has more than one hundred plant species as host, which leads towards momentous economic losses [8].

Mineral nutrients (macro and micro) play a crucial role in the life of chilli plants as their deficiency or excess amount have deleterious effects on plants [9], which makes the host plants vulnerable to pathogen attack. Being an integral part of plant metabolism, nutritional deficiency incited various diseases [10] by disturbing plant metabolism and physiology [11]. Excess amounts of nutrients cause toxicity in plants [12], leading towards the accumulation of sugar and carbohydrates. Higher amount of sugar and carbohydrates enhance susceptibility by facilitating the attack of many pathogens, which indicates the direct involvement of mineral nutrients in defense mechanism of host in the form of metabolic regulators and structural components [13]. Nutritional status being one of the first line of defense of the host plants, determines their susceptibility or resistance against invading pathogens [10]. When pathogen attacks, it feed on the nutrients of host plants [14]. Due to this, host plants can’t meet their nutritional requirement and become weaker, and a weak plant can’t properly run its normal physiological and defense functions, including the activation of defense system against invading pathogens.

During normal metabolic process, highly reactive compounds (free radicals) are produced in the form of reactive oxygen species (ROS) in plants [15]. The balanced concentration of these free radicals (ROS) is beneficial for plant’s indispensable processes, such as the activation of defense through intracellular signaling against pathogens [16]. Too high concentration of ROS is lethal for plants; therefore, ROS needs to be stabilized naturally by various antioxidant enzymes (superoxide dismutase, glutathione peroxidase, and catalase) present in plants [17]. Collectively, these biochemical compounds improve plant health and make their considerable contribution in the activation of defense system [18, 19] by producing different types of enzymes and metabolites. Attacks of pathogens cause disturbance or alterations in these free radicals and antioxidant enzymes of plants [20]. These biochemical alterations induced by pathogens lead to the host plant towards abnormality by disturbing physiological processes [21, 22]. The disturbed biochemical profile of plants can be improved by the application of different macro- and micro- nutrients. Nutrients actively function as metabolic regulators, contributing to enhanced plant health and the improvement of the plant’s biochemical profile [23]. Moreover, nutrients stimulate the production of essential secondary metabolites, such as phenolics, alkaloids, and terpenoids. Micronutrients like Se, Mn, and Zn contribute to the synthesis and functioning of superoxide dismutase, glutathione peroxidase, and catalase, while Ca acts as a secondary messenger in signaling pathways. Considering the gravity of the issue, the current study aimed to determine the biochemical compounds in both nutrient product-treated and untreated chili plants. Determination of biochemical compounds is helpful for scientists towards the development of biochemical markers for identifying resistant sources in chilli plants. Moreover, these biochemical analyses assist the scientists and researchers to understand the mechanism of resistance and susceptibility towards pathogens, which is also helpful in the product development to manage the Fusarium wilt of chilli and potentially other pathogens. We hypnotized that mineral nutrients can be very helpful in managing Fusarium wilt disease of chilli by boosting plant defense through their direct impact on various antioxidant defensive enzymes. Therefore, the objective of this present study was to investigate the management of Fusarium wilt of chilli through diverse mineral nutrients by boosting plant defense.

## 2. Materials and methods

### 2.1 Material collection

All chemical and reagents used were of analytical grade, for instance, potassium dihydrogen phosphate (KH_2_PO_4_), silver nitrate, metaphosphoric acid, Hydrochloric acid, dinitrosalicylic acid, and Sodium Nitrite were purchased from Sigma Chemical Co. (St. Louis, Mo, USA. Ptoassium iodide, dichloroindophenol, and trichloroacetic acid were purchased from Merck (Darmstadt, Germany). Potassium Chloride, Di potassium hydrogen phosphate, and EDTA were purchased from Samchun. Aluminum chloride and Catechin were purchased from PubChem. Acetone, F-C reagent, and Sodium Hydroxide were purchased from Riedel-de Haen (Germany), Oxford lab chem (India), and Merck Millipore (USA), respectively.

### 2.2 Experimental setup (*in vitro*)

Moderately susceptible variety of chilli (Hot Shot) plants were grown in germination trays (200 holes trays with 2–2’’ hole size) containing peat moss media (2–2.5 g/hole). After forty days, the seedlings were ready to be transplanted. Plants (4–6 leaf stage) were transferred in pots (17×13Cm) containing formalin sterilized sandy loam soil (60% Sand, 10% Clay, and 30% Silt). After one week, two nutrient fertilizers Krystafeed (Arysta Life Science) (T1), Micro Plus (Dalton^™^) (T2), and their combinations (T3) were applied by soil drenching method using glass beaker at three different concentrations (Krystafeed @ (C1) 2, (C2) 4, and (C3) 6g/liter/Pot; Micro Plus @ (C1) 0.5, (C2) 0.75, and (C3) 1.0g/liter of water/pot; combination of Krystafeed and Micro Plus @ (C1) 2+0.5, (C2) 4+0.75, and (C3) 6+1.0g/liter/pot, respectively). Control treatment was maintained by applying distilled water on chilli plants. The experiment was performed in three replicates. The spore suspension was prepared from a one-week-old culture exhibiting sporulation and dense growth of Fusarium mycelium. Macro and micro conidia were collected by scraping the fungal mass and dissolving it in distilled water. Spore suspension of *Foc* was measured and diluted to 1×10^6^ spores/mL with the help of a hemocytometer (SKU:100158). Inoculation was done early in the morning by soil drenching method [24]. For this purpose, 100mL of spore suspension was applied in the root zone or near the base of the plant by digging 2–3 cm holes to provide maximum inoculum pressure. Data regarding disease incidence was recorded by using Moniam and Ismail scale [25]. According to this scale 0 = immure, 1 = 1–20% resistant, 2 = 21–40% moderately resistant, 3 = 41–50% moderately susceptible, 4 = 51–70% susceptible, and 5 = 70–100% highly susceptible.

### 2.3 Experimental setup (*in vivo*)

For the evaluation of macro- and micro- nutrients in field, two nutrient products Krystafeed (T1), Micro Plus (T2), and their combinations (T3) (Table 1) were mixed at three different concentrations (Krystafeed @ (C1) 300, (C2) 350, and (C3) 400g/100ft^2^; Micro Plus @ (C1) 30, (C2) 35, and (C3) 40 g/100ft^2^; combination of Krystafeed and Micro Plus @ (C1) 300+30, (C2) 350+35, (C3) 400+40 g/100ft^2^, respectively) in sandy loam soil sterilized with 5% formalin solution. Plants (4–6 leaf stage) of moderately susceptible variety (Hot Shot) of chilli were transferred in field under RCBD by maintaining three replications. Chilli plants were treated with the *Foc* suspension (1×10^6^ spores/mL of water) by soil drenching method to give them the proper inoculum pressure [24]. The data regarding disease incidence was recorded after one weak interval. Disease incidence data was recorded by following formula:

$$\text{Disease}\;\text{incidence}\;\left(\text{\%}\right)=\frac{\text{No}.\;\text{of}\;\text{infected}\;\text{Plants}}{\text{Total}\;\text{no}.\;\text{of}\;\text{observed}\;\text{Plants}}\times 100$$

**Table 1:** Composition of Krystafeed and Micro Plus used against Fusarium wilt of chilli.

Nutrient Fertilizer (%)	Krystafeed (25kg/bag)		Micro Plus (1 Kg/Pack)	
	% percentage	g/bag	% percentage	g/Pack
Nitrogen (N)	15	3750	-	-
Phosporus (P)	18	4500	-	-
Potassium (K)	18	4500	-	-
Iron (Fe)	-	-	15%	150
Magnesium (Mg)	-	-	11%	110
Manganese (Mn)	-	-	2.4%	24
Copper (Cu)	-	-	1%	10
Zinc (Zn)	-	-	1%	10
Boron (B)	-	-	0.2%	2
Molybdenum (Mo)	-	-	0.04%	0.4

### 2.4 Sample preparation for biochemical profiling

For the biochemical profiling, leaf samples were collected from both groups, for example, the treated (most effective/best performed treatment) and untreated (application of distilled water) ones. The leaf samples were collected early in the morning of the 7^th^ day of inoculation from top, middle, and lower portions of the plants (6–8 leaf stage) for the *in-vitro* experiment. The samples were made dirt free by washing with tap water and then with distilled water. After this, leaves were air dried to remove extra water contents from surface and cut into small pieces (1-2mm). Then 0.5 grams of sample (leaves) was mixed in the extraction buffer (10mM K_2_HPO_4_, 10mM KH_2_PO_4_, 100mM KCL, and 2mM EDTA) at pH 7 with 1: 2 ratios. The mixture was grinded with the help of sterilized pestles and mortars and filled in Eppendorf tubes (1.5 mL), which was centrifuged (TGL-16A) at 12,000rpm for 10 minutes. Supernatant was taken only and stored at -20°C by using sterilized bottles in refrigerator (PEL, PRGD-145) for further biochemical analysis.

#### 2.4.1 Measurement of the level of superoxide dismutase (SOD)

Reaction mixture was prepared by using different chemicals, including 200μL methionine (C_5_H_11_N_2_S), 100μL NBT (Nitro-blue Tetrazolium), 100μL enzyme extract, potassium phosphate buffer, triton X 500μL, and 800μL distilled water. This reaction mixture was poured into a test tube (10mL) and placed under ultraviolet radiation (light intensity 350 μmol m^-2^ s^-1^, UV Transilluminator SKU: E3000) for fifteen minutes. After adding 100μL riboflavin, the absorbance was taken at 560nm by using an ELISA plate reader [26]. The amount of superoxide dismutase was calculated as the quantity of enzyme needed to induce a 50% reduction in Nitroblue Tetrazolium activity.

#### 2.4.2 Test of the activities of Peroxidase (POD) and Catalase (CAT)

To determine peroxidase from chilli leaves, the reaction mixture was prepared by adding 100μL H_2_O_2_, 100μL enzyme extract, 800μL potassium phosphate buffer (pH 5), and 20mM guaiacol into leaf extract in a 2mL tube. Absorbance was noted at 470nm by using the spectrophotometer (Hitachi U-2001, model 121–003). The reaction mixture for the determination of CAT was prepared by adding 100μL enzyme extract and 100μL H_2_O_2_. Spectrophotometer (Hitachi U-2001, model 121–003) was used to measure the absorbance at 240nm [27]. Peroxidase and catalases were determined by following formulas:

$$Peroxidase\;\left(\mu \text{g}/\text{g}\right)=\frac{A_{470}\times 1000}{43.6}$$

$$Catalase\;\left(\mu \text{g}/\text{g}\right)=\frac{A_{240}\times 1000}{43.6}$$

#### 2.4.3 Measurement of H_2_O_2_ concentration

For the determination of H_2_O_2_, 0.5g leaf sample was taken and grinded in 0.1% Tri-chloro-acetic acid (C_2_HCl_3_O_2_), using pestles and mortars. This mixture was centrifuged at 12,000 rpm for 15 minutes. Supernatant was separated in the Eppendorf tubes (1.5mL) and pellets were discarded. Supernatant was dissolved in 1.3mL potassium phosphate buffer (pH 7) and 1mLpotassium iodide in a test tube (5mL). The reaction mixture was incubated for 5 min and the absorbance was recorded at 390 nm by using the absorbance reader (BioTek, Model: 800TS). The amount of H_2_O_2_ was calculated by following formula [28].


$$\text{Hydrogen}\;\text{peroxide}\;\left(\mu \text{g}/\text{g}\right)=\frac{Blank\;Absorbance-Sample\;Absorbance}{Blank\;Absarbance}\times 100$$

#### 2.4.4 Test of the total phenolic contents

Ten mL of extraction mixture and 1g of grinded sample was dissolved in test tubes (15mL). This mixture was vortexed and centrifuged for five minutes. Then 100μL supernatant was separated and added into 200μL F-C reagents (10%) and vortexed. 800 μL Na_2_CO_3_ was added into it, vortexed, and left for one hour. 200μL solution was loaded in one ELISA plate and absorbance was recorded at 765 nm [29].


$$\text{Total}\;\text{Phenolic}\;\text{Contents}\;\left(\mu \text{g}/\text{mL}\right)=\frac{Sample\;Absorbance-Blank\;Absorbance}{0.005}\times \;\frac{1}{1}$$

#### 2.4.5 Determination of chlorophyll contents

To determine chlorophyll contents, fresh leaves of chilli were collected, grinded, and extracted in acetone (80% solution). Centrifugation was done at 12,000 rpm for 5 minutes to purify the enzyme extract. The supernatant was separated, and the absorbance was taken by a spectrophotometer (Hitachi U-2001, model 121–003) at 645, 663, and 480 nm [30, 31]. Whereas the chlorophyll contents were determined by following formulas:

$$\text{Total}\;\text{Chlorophyll}\;\left(\text{mg}/\text{g}\right)=20.20A_{663}+8.02A_{645}$$

$$\text{Chlorophyll}\;a\;\left(\text{mg}/\text{g}\right)=12.7A_{663}-2.69A_{645}$$

$$\text{Chlorophyll}\;b\;\left(\text{mg}/\text{g}\right)=22.9A_{645}-4.68A_{663}$$

#### 2.4.6 Estimation the level of tocopherol (μg/mL)

Estimation of the level of tocopherol was carried out by adding 1 mL of pure ethanol into 4 mL test tubes. 0.2 mL of bathophenanthroline (0.2%) and 0.2 mL of Ferric Chloride FeCl_3_ (0.001M) was added into ethanol containing test tubes and mixed thoroughly using a vortex mixer (Velp Scientifica SA202A0176 - ZX3). After a pause of 60 seconds, the addition of 0.2 mL phosphoric acid H_3_PO_4_ (0.001M) was done. The reaction mixture was mixed thoroughly, and the absorbance was taken at 534 nm with the help of a spectrophotometer [32]. The amount of tocopherol was taken in μg/mL and was calculated by using standard curve.

#### 2.4.7 Assessment of the level of thioredoxin reductase

For the assessment of Thioredoxin Reductase (TrxR), the reaction mixture was prepared by adding 200 μL NADPH, 10 mL ETDA, 5 mL 5,5’-Dithiobis (2-nitrobenzoic acid) (DTNB), and 30 μL enzyme extract into 100 mL of potassium phosphate buffer (pH 7.0). Reaction mixture was divided into two groups. 10 μL of TrxR Inhibitor was added in one group for testing the background enzyme activity; and 10 μL of Assay Buffer was added into 2nd group for estimating the total DTNB reduction. Absorbance of these reaction mixtures was taken at 412 nm [33]. TrxR activity was measured in nmolmin^-1^ml^-1^ after preparing TNB standard curve.

$$\text{TrxR}\;\text{Activity}=\frac{\Delta \text{B}}{\left(T2-T1\right)\times \text{V}}\times \text{Sample}\;\text{Dilution}\;\text{Factor}=\text{mU}/\text{mL}$$

Where:

ΔB = TNB amount from TNB standard curve in nmol

T_2_ = Time of second reading (in min)

T_1_ = Time of first reading (in min)

V = pretreated sample volume (ml) added into the reaction well

#### 2.4.8 Estimation of the level of Malondialdehyde (MDA)

Cakmak and Horst [34] method was used for the estimation of the level of MDA (Malondialdehyde). For this purpose, 1g of leaf samples were ground in 20 mL of trichloroacetic acid (0.1%). After homogenization, this solution was poured into eppendorf tubes (1.5 mL) and centrifuged at 12000 rpm for 10 minutes. 4 mL of mixed solution of trichloroacetic acid (20%) and thiobarbituric acid (0.5%) was taken and added into 1 mL of supernatant. This solution was heated at 100 °C for 30 minutes in water bath (Model: DK-98-IIA; Dimensions: 355×240×190mm) and then suddenly cooled down on ice bath (Model: CB-200D-IB Electronic Ice Bucket; Dimensions: 190 x 240 x 225 mm). This solution was centrifuged at 12,000 rpm for 10 minutes and supernatant was separated to take the absorbance at 532 & 600 nm. The amount of MDA was determined by using the extinction coefficient of 155/(mM/cm) using the relation shown in equation below, where A is the absorbance.


$$MDA\;Level\;\left(nM\right)=\frac{\Delta \left(A532nm-A600nm\right)}{1.56}\times 105$$

#### 2.4.9 Estimation of the level of ascorbate

Estimation of ascorbate level was taken by mixing 100 μL metaphosphoric acid (0.1%), 100 μL enzyme extract, and 1 mL dichloroindophenol into 900 μL distilled water. The solution was mixed well using vortex (Velp Scientifica SA202A0176 - ZX3), 200 μL/sample was loaded on 96 well ELISA plate and absorbance was taken at 520 nm [35]. The amount of ascorbate was taken as μg/mL.

#### 2.4.10 Estimation of proline contents

For the estimation of proline contents, 0.1 g leaf plant samples were taken and homogenized into 5mL of 5-sulfosalicylic acid (3%). Acid ninhydrin was prepared by dissolving 1.25 g of ninhydrin into 20 mL of phosphoric acid (6 M) and 30 mL of glacial acetic acid. The plant extract was reacted with 2 mL acid ninhydrin and 2 mL glacial acetic acid at 100 °C for 60 minutes. This reaction was ended in ice bath. After the addition of 1 mL toluene, the absorbance was recorded at 520 nm [36]. Proline contents were determined by following equation:

$$Proline\;\left(\mu \text{mole}.{\text{g}}^{-1}\right)=\frac{A_{520}-A_{Blank}}{Slope}*\;\frac{Vol_{extract}}{Vol_{aliquot}}*\;\frac{1}{FW}$$

Where:

A_520_ = Sample absorbance at 520nm absorbance

Slope = Determined by linear regression (expressed as absorbance∙nmol^-1^)

Vol_extract_ = Total volume of the extract

Vol_aliquot_ = Volume used in the assay

FW = Amount of plant material extracted (expressed in mg)

#### 2.4.11 Measurement of the phytoalexin (Capsidiol) level

Color reaction in thin layer chromatography was used to identify the phytoalexin (Capsidiol). Pure capsidiol (known concentrations) was mixed with ethyl acetate and poured into eppendorf tubes (1.5 mL). After the evaporation of ethyl acetate, residue was redissolved into ethanol absolute (0.5 mL) with vertexing. 0.5 mL of vanillin (2%) was gently added into each eppendorf tubes and vortexed carefully. These tubes were incubated for 10 minutes at the room temperature. Color reactions produced by the different amounts of capsidiol were analyzed by taking absorbance at 200–700 nm by using a spectrophotometer [37].

### 2.5 Statistical analysis

Analysis of variance (ANOVA) was used to test the effects of different treatments on disease incidence of Fusarium wilt and biochemical attributes of chilli plants with the least significant difference (LSD) at p<0.05 using Statistics 8.1 software. Moreover, Pearson correlation analysis between various enzymes of antioxidant defense system was performed in OriginPro 2023.

### 2.6 Computational analysis of selected enzymes for biochemical metabolism

The complete protein sequences of the superoxide dismutase (NP_001311927.1), peroxidase (AAL35364.1), catalase (NP_001311603.1), chitinase (ACM47315.1), and ribulose-1,5-bisphosphate carboxylase/oxygenase (AID55408.1) metabolic enzymes from *Capsicum annuum* were obtained from the NCBI GenBank database (https://www.ncbi.nlm.nih.gov). The 3D models of the chosen metabolic enzymes were acquired from the online Alpha Fold Protein Structure Database, a resource jointly developed by EMBL-EBI and DeepMind (https://alphafold.ebi.ac.uk/) [38]. The PDB files for the chosen metabolic enzyme models were accessed, and the 3D representations of these selected metabolic enzymes from the *Capsicum annuum* were visualized using the freely accessible Discovery Studio software (https://discover.3ds.com/discovery-studio-visualizer-download). The precision of the metabolic enzyme models in the *Capsicum annuum* was verified using the freely accessible SAVES server available online which has been used to study cytochrome b protein ((https://saves.mbi.ucla.edu/) [39]. The ERRAT server was used to establish the overall quality factor for the non-bonded atomic interactions in all the chosen metabolic enzyme models in the *Capsicum annuum* (https://servicesn.mbi.ucla.edu/ERRAT/) [40]. The PROCHECK server was employed to predict the stereochemical characteristics of the overall structural geometry of the chosen metabolic enzyme models in the *Capsicum annuum* with reference to amylase studies in different model organisms (https://servicesn.mbi.ucla.edu/PROCHECK/) [41, 42]. The interactions between proteins and distinct metal ions play a pivotal role in various physiological processes. MIB2 (metal ion-binding) strives to address the constraint by structure-based prediction methods, as numerous proteins still lack resolved structures [43]. Nine metal ions (Ca^2+^, Cu^2+^, Fe^3+^, Mg^2+^, Mn^2+^, Zn^2+^, Ni^2+^, Co^2+^, and Ba^2+^) which have major and minor contributions in plants’ nutrition were extensively docked with five selected metabolic enzymes to find their suitable binding sites [44]. Moreover, MIB2 provides enhanced predictive accuracy and encompasses a broader range of metal ion types (http://combio.life.nctu.edu.tw/MIB2/) [45].

## 3. Results

### 3.1 Impact of plant nutrients on disease incidence

**Fig 1:**
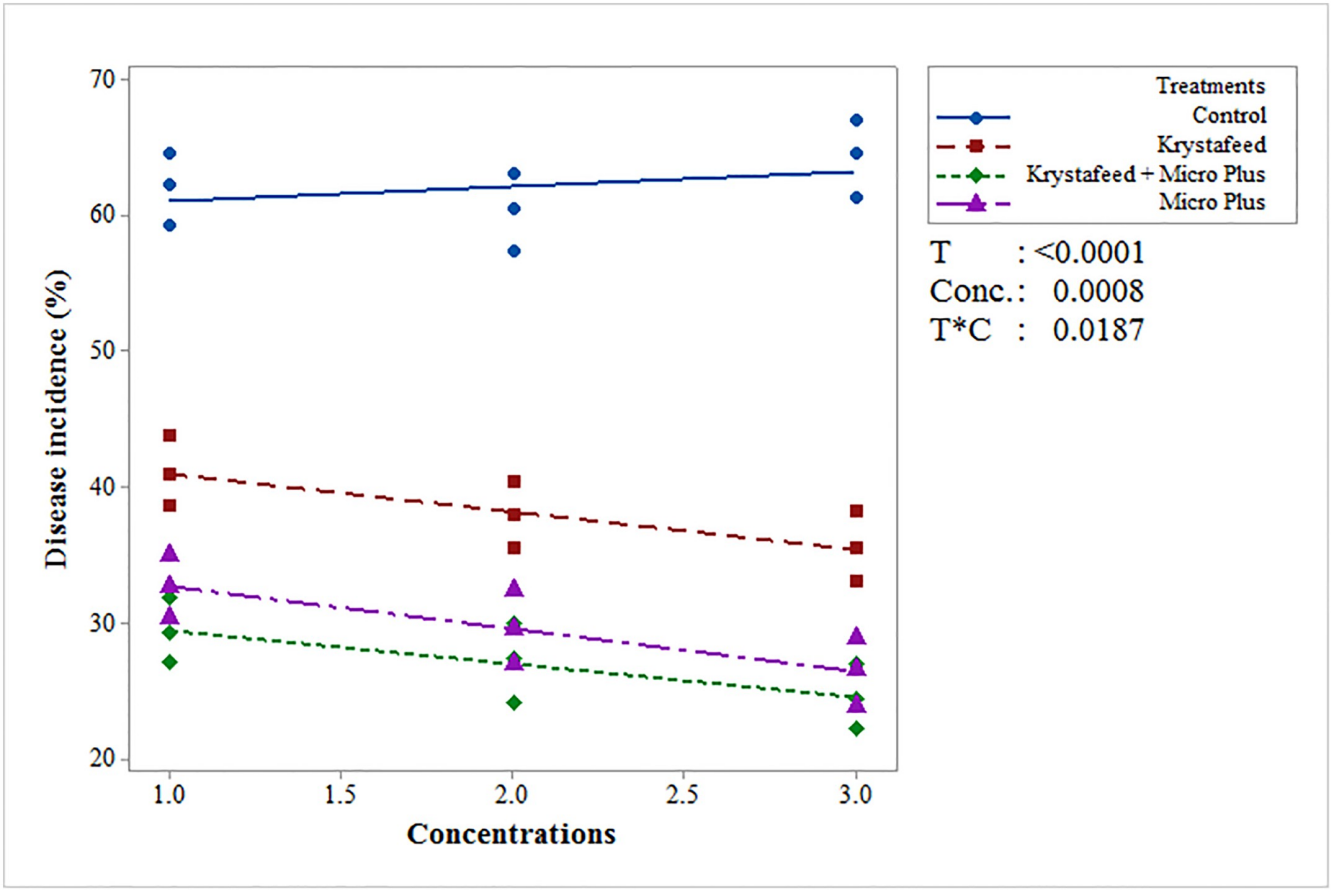
Effect of different plant nutrients on the incidence of Fusarium wilt of chilli under greenhouse conditions at three different concentrations (Krystafeed @ (C1) 2, (C2) 4, and (C3) 6g/liter/Pot; Micro Plus @ (C1) 0.5, (C2) 0.75, and (C3) 1.0g/liter of water/pot; Combination of Krystafeed and Micro Plus @ (C1) 2+0.5, (C2) 4+0.75, and (C3) 6+1.0g/liter/pot). The P-values for treatments (T), concentrations (C), and their interaction (T*C) showed significant statistical differences (p<0.05) among them.

**Fig 2:**
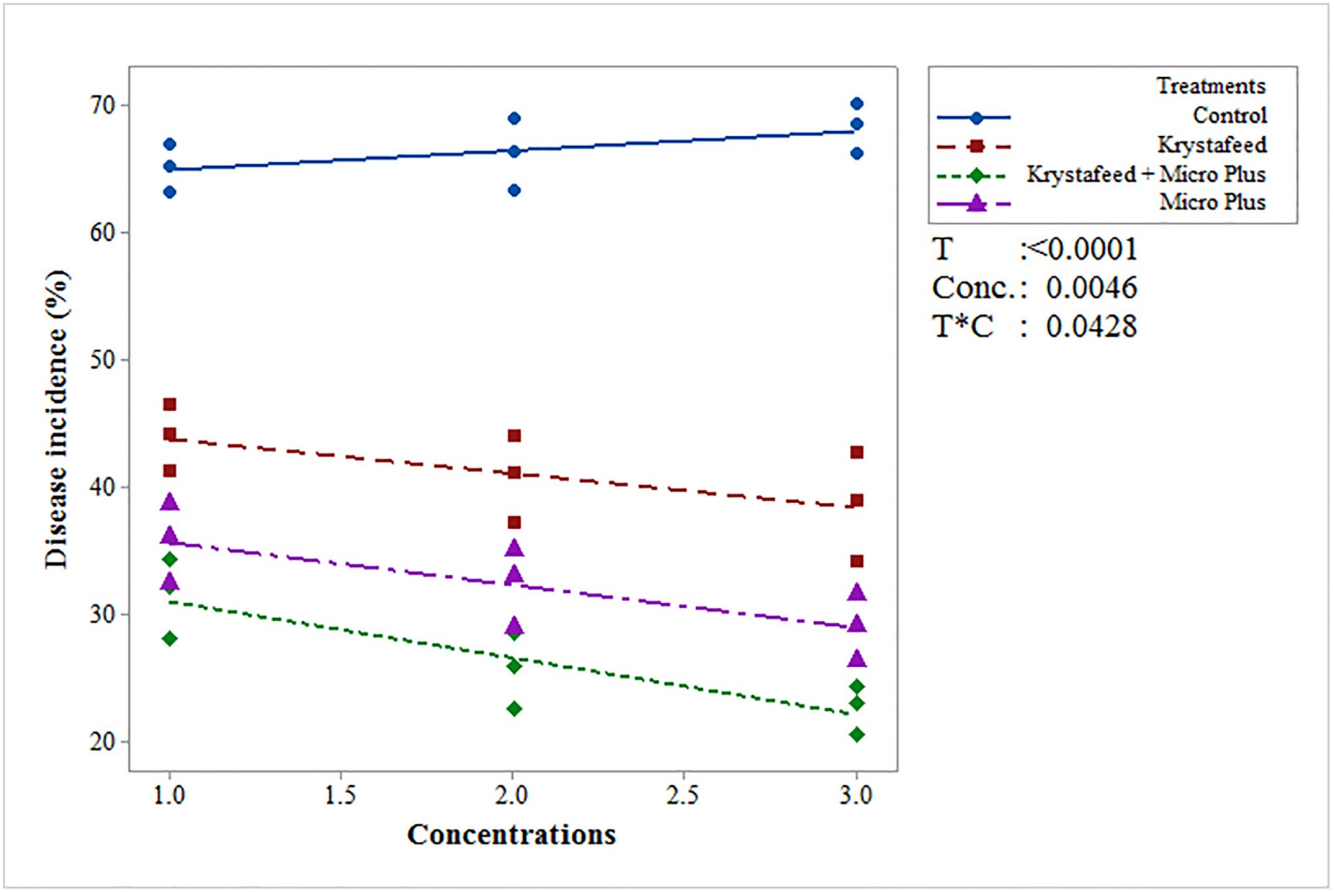
Effect of different plant nutrients on the incidence of Fusarium wilt of chilli under field conditions at three different concentrations (Krystafeed @ (C1) 300, (C2) 350, and (C3) 400g/100ft^2^; Micro Plus @ (C1) 30, (C2) 35, and (C3) 40 g/100ft^2^; Combination of Krystafeed and Micro Plus @ (C1) 300+30, (C2) 350+35, and (C3) 400+40 g/100ft^2^). The P-values for treatments (T), concentrations (C), and their interaction (T*C) showed significant statistical differences (p<0.05) among them.

Among evaluated plant nutrients, the combination of Krystafeed and Micro Plus (T_3_) exhibited minimum disease incidence under greenhouse and field conditions followed by Micro Plus (T_2_) and Krystafeed (T_1_) as compared to the control (Fig 1). In case of interaction between treatments and concentrations (T × C), the combination of Krystafeed and Micro Plus showed minimum disease incidence (29.41, 27.13, 24.51%) at C_1_, C_2_ and C_3_, concentrations respectively, followed by Micro Plus (32.66, 29.6, 26.43%) and Krystafeed (41.12, 37.96, 35.57%) as compared to the control under greenhouse conditions. While under field conditions, the interaction between T × C showed that T_3_ exhibited significant results with least disease incidence (31.52, 25.62, 22.63%) followed by T_2_ (35.76, 32.33, 28.98%) and T_1_ (44.01, 40.81, 38.63%) as compared to the control (Fig 2).

### 3.2 Impact of plant nutrients on biochemical compounds

#### 3.2.1 Activity of SOD, POD, CAT, and H_2_O_2_

**Fig 3:**
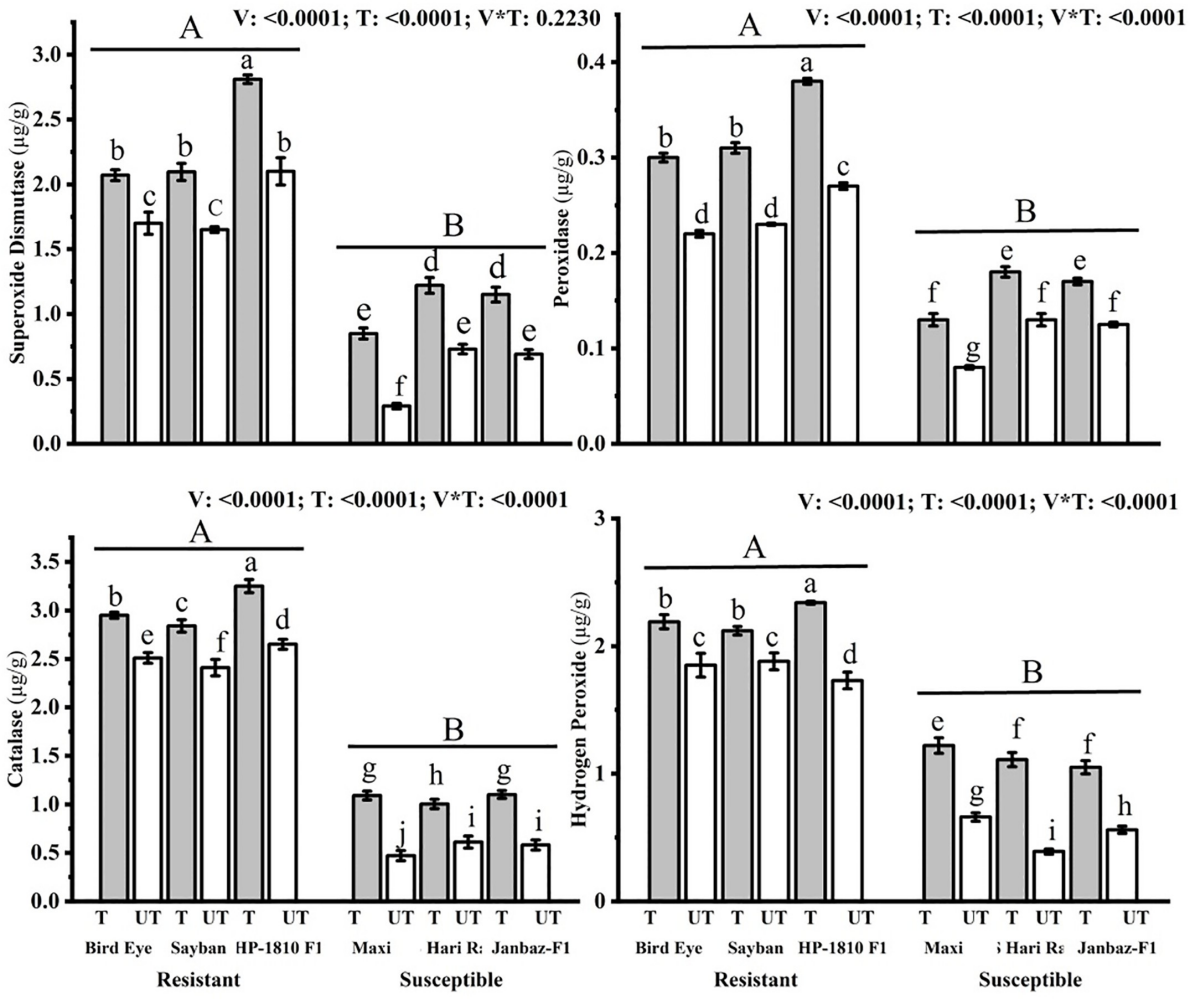
Effect of plant nutrients on the levels of the superoxide dismutase (μg/g), peroxidases (μg/g), catalase (μg/g), and hydrogen peroxide (μg/g) in different treated (T) and untreated (UT) plants of chilli. Small letters define the statistical significance (p<0.05) in different treated (T) and Un-treated (UT) plants. Capital letters indicate significant difference (p<0.05) among the resistant and susceptible varieties of chilli.

All biochemical enzymes, including superoxide dismutase, peroxidase, catalase, and hydrogen peroxide, were significantly increased in the treated (T) plants of resistant (Bird eye, Sayban, HP-1810 F1) and susceptible (Maxi, BS Hari Rani, Janbaz-F1) varieties of chilli (Fig 3). Application of most effective treatment T_3_ at C_3_ significantly (p<0.05) increased levels of the superoxide dismutase (0.37, 0.45, 0.71, 0.56, 0.49, 0.46)μg/g, peroxidase (0.08, 0.08, 0.11, 0.05, 0.05, 0.045)μg/g, catalase (0.44, 0.43, 0.6, 0.62, 0.393, 0.52)μg/g, and hydrogen peroxide (0.34, 0.24, 0.61, 0.56, 0.72, 0.49)μg/g in the treated plants of chilli varieties, such as Maxi, BS Hari Rani, Janbaz-F1, Bird eye, and Sayban, HP-1810 F1, respectively, as compared to the untreated plants (Fig 3).

#### 3.2.2 Impact on phenolic contents and photosynthetic pigments

**Fig 4:**
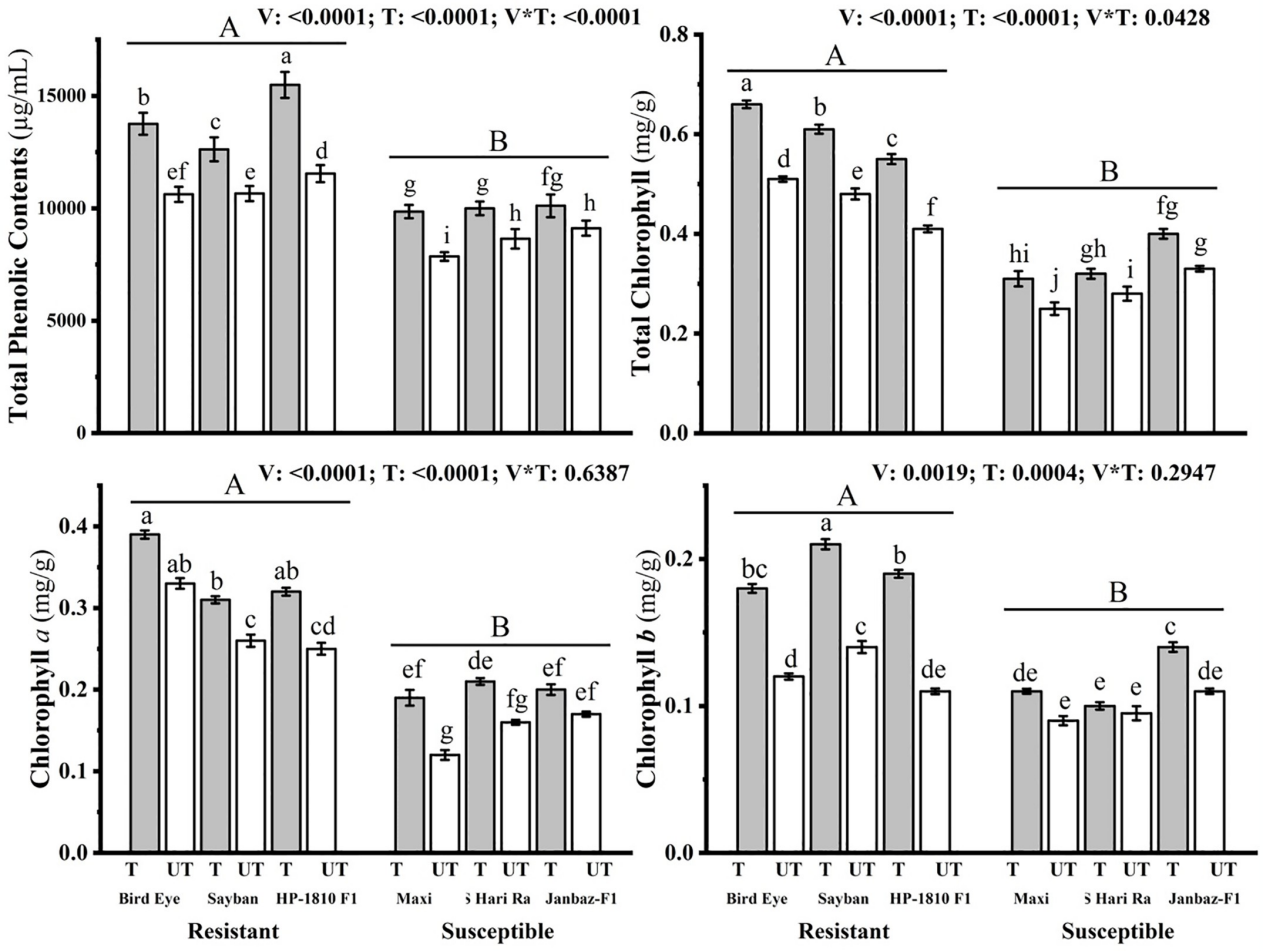
Effect of plant nutrients on the total phenolic contents (μg/mL), total chlorophyll (mg/g), chlorophyll *a* (mg/g), and chlorophyll *b* (mg/g) in different treated (T) and untreated (UT) plants of chilli. Small letters define the statistical significance (p<0.05) in different treated (T) and Un-treated (UT) varieties. Capital letters indicate significant difference (p<0.05) among resistant and susceptible varieties of chilli.

Total phenolic contents, chlorophyll *a*, chlorophyll *b*, and total chlorophyll were significantly (p<0.05) increased in the treated plants (T_3_ at C_3_ concentration) of resistant varieties (Bird eye, Sayban, HP-1810 F1) by (29.51, 18.48, 34.18%), (18.18, 19.23, 28%), (50, 50, 72.72%) and (29.41, 27.08, 34.14%), respectively, as compared to the untreated plants. While the amount of TPC, Chl *a*, Chl *b*, and total Chl was significantly increased in the treated plants of susceptible varieties (Maxi, BS Hari Rani, Janbaz-F1) by (25.44, 15.71, 10.97%), (58.33, 31.25, 17.64%), (22.22, 5.26, 27.27%), and (24, 14.28, 21.21%), respectively as compared to the untreated plants (Fig 4).

#### 3.2.3 Activity of Tocophherol, TrxR, MDA, ascorbate

**Fig 5:**
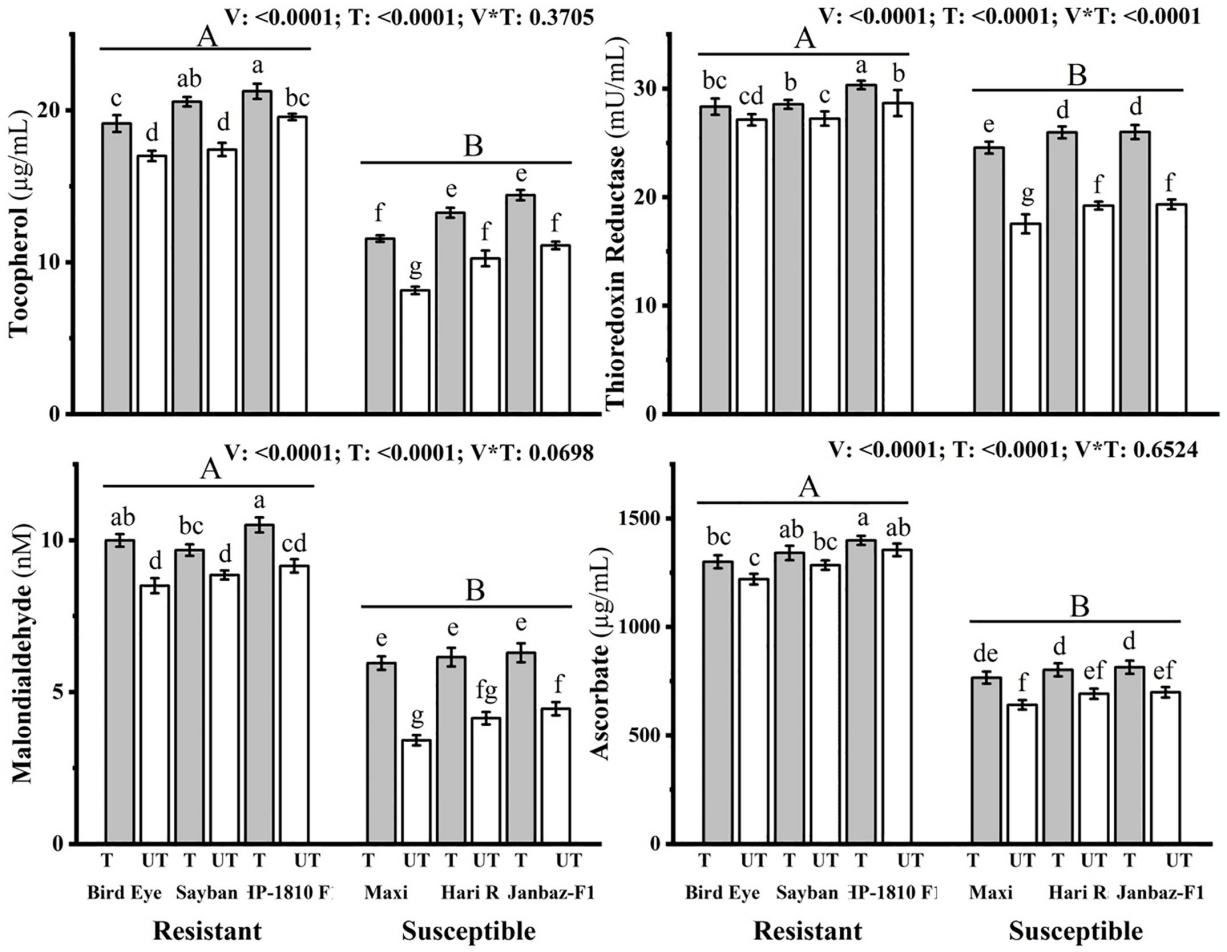
Effect of plant nutrients on tocopherol (μg/mL), thioredoxin reductase (mU/mL), malondialdehyde (nM), and ascorbate (μg/mL) in different treated (T) and untreated (UT) plants of chilli. Small letters define the statistical significance (p<0.05) in different treated (T) and Un-treated (UT) plants of chilli. Capital letters indicate significant differences (p<0.05) among resistant and susceptible varieties of chilli.

Application of the most effective treatment (T3) significantly (p<0.05) increased tocopherol (2.13, 3.15, 1.69), (3.41, 3, 3.31)μg/mL, thioredoxin reductase (1.21, 1.31, 1.67), (7.03, 6.75, 6.66) mU/mL, malondialdehyde (1.49, 0.82, 1.35), (2.54, 2.01, 1.84) nM, and ascorbate (80, 56, 44), (125, 110, 115)μg/mL in the treated (T) plants as compared to the untreated (UT) plants of resistant (Bird eye, Sayban, HP-1810 F1) and susceptible (Maxi, BS Hari Rani, Janbaz-F1) chilli varieties, respectively (Fig 5).

#### 3.2.4 Impact on proline and capsidiol contents

**Fig 6:**
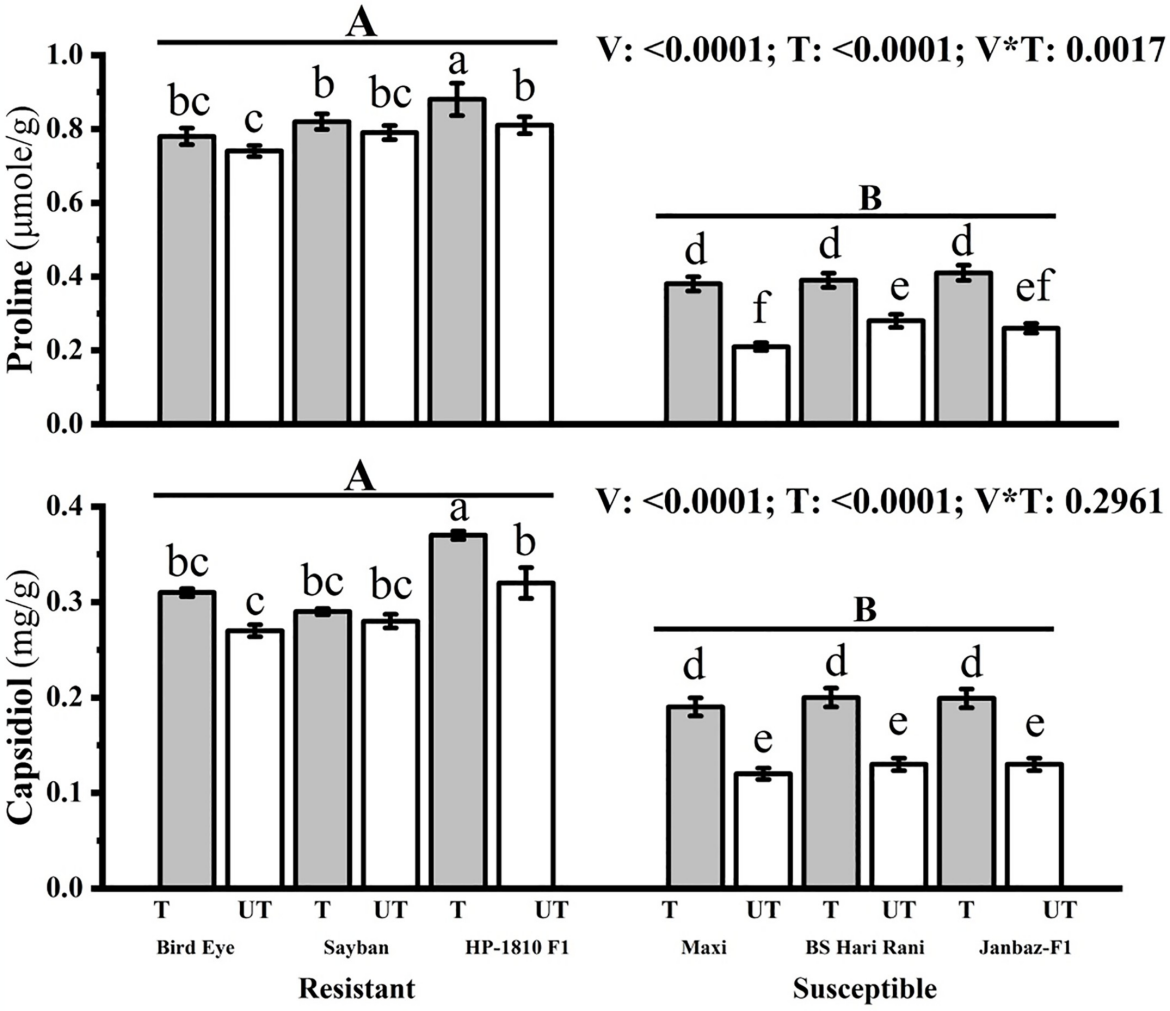
Effect of plant nutrients on proline (μmole/g) and phytoalexin (capsidiol) (mg/g) in different treated (T) and untreated (UT) plants of chilli. Small letters define the statistical significance (p<0.05) in different treated (T) and Un-treated (UT) varieties. Capital letters indicate significant difference (p<0.05) among resistant and susceptible varieties of chilli.

The proline contents of resistant varieties (Bird Eye, Sayban, HP-1810 F1) significantly increased by 5.40%, 3.79%, and 8.64%, respectively, in treated plants compared to untreated ones. Similarly, susceptible varieties (Maxi, BS Hari Rani, Janbaz-F1) showed significant increases of 80.95%, 39.28%, and 57.69%, respectively, in proline content in treated plants compared to untreated plants (Fig 6). The concentration of capsidiol increased by 14.81%, 3.57%, and 15.62% in treated plants of resistant varieties (Bird Eye, Sayban, HP-1810 F1), respectively. In contrast, the increase in treated plants of susceptible varieties (Maxi, BS Hari Rani, Janbaz-F1) was 58.33%, 53.84%, and 53.07%, respectively, compared to untreated plants (Fig 6).

### 3.3 Correlation analysis

**Fig 7:**
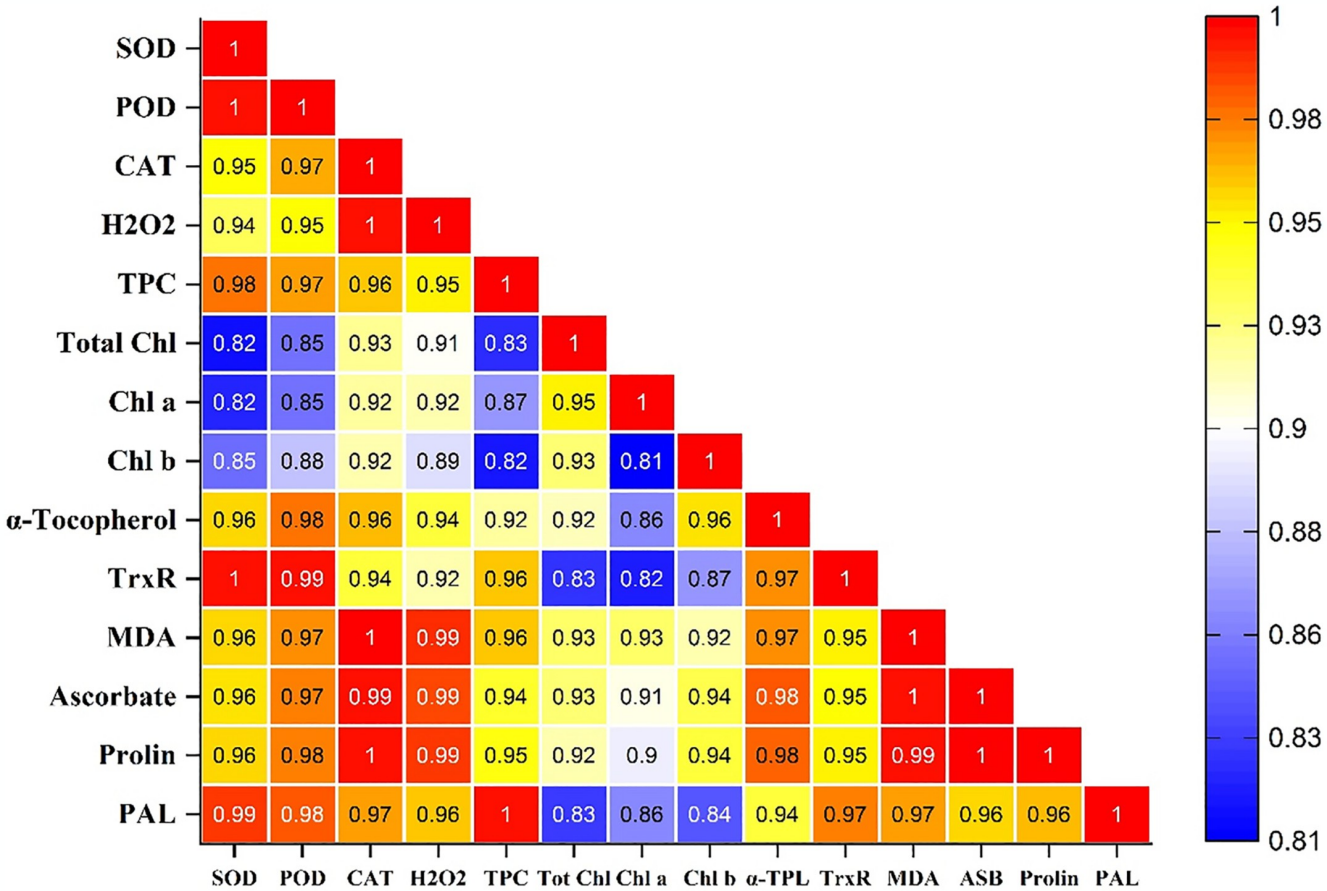
Pearson’s correlation heatmap analysis between various enzymes of antioxidant defense system where SOD = Superoxide Dismutase; POD = Peroxidases; CAT = Catalases; H_2_O_2_ = Hydrogen Peroxide; TPC = Total Phenolic Contents; Chl = Chlorophyll; TrxR = Thioredoxin reductase; MDA = Malondialdehyde; PAL = Phytoalexin (Capsidiol).

The Pearson correlation analysis showed that there is a strong correlation exists between various enzymes of antioxidant defense system (Fig 7). Superoxide dismutase was positively correlated with peroxidases (r = 0.99), catalases (r = 0.95), hydrogen peroxide (r = 0.93), total phenolic contents (r = 0.97), total chlorophyll (r = 0.81), chlorophyll *a* (r = 0.82), chlorophyll *b* (r = 0.84), α-Tocopherol (r = 0.95), TrxR (r = 0.99), MDA (r = 0.95), ascorbate (r = 0.95), prolin (r = 0.95), and phytoalexin (r = 0.98).

### 3.4 Computationally exploration of metabolic enzymes

**Fig 8:**
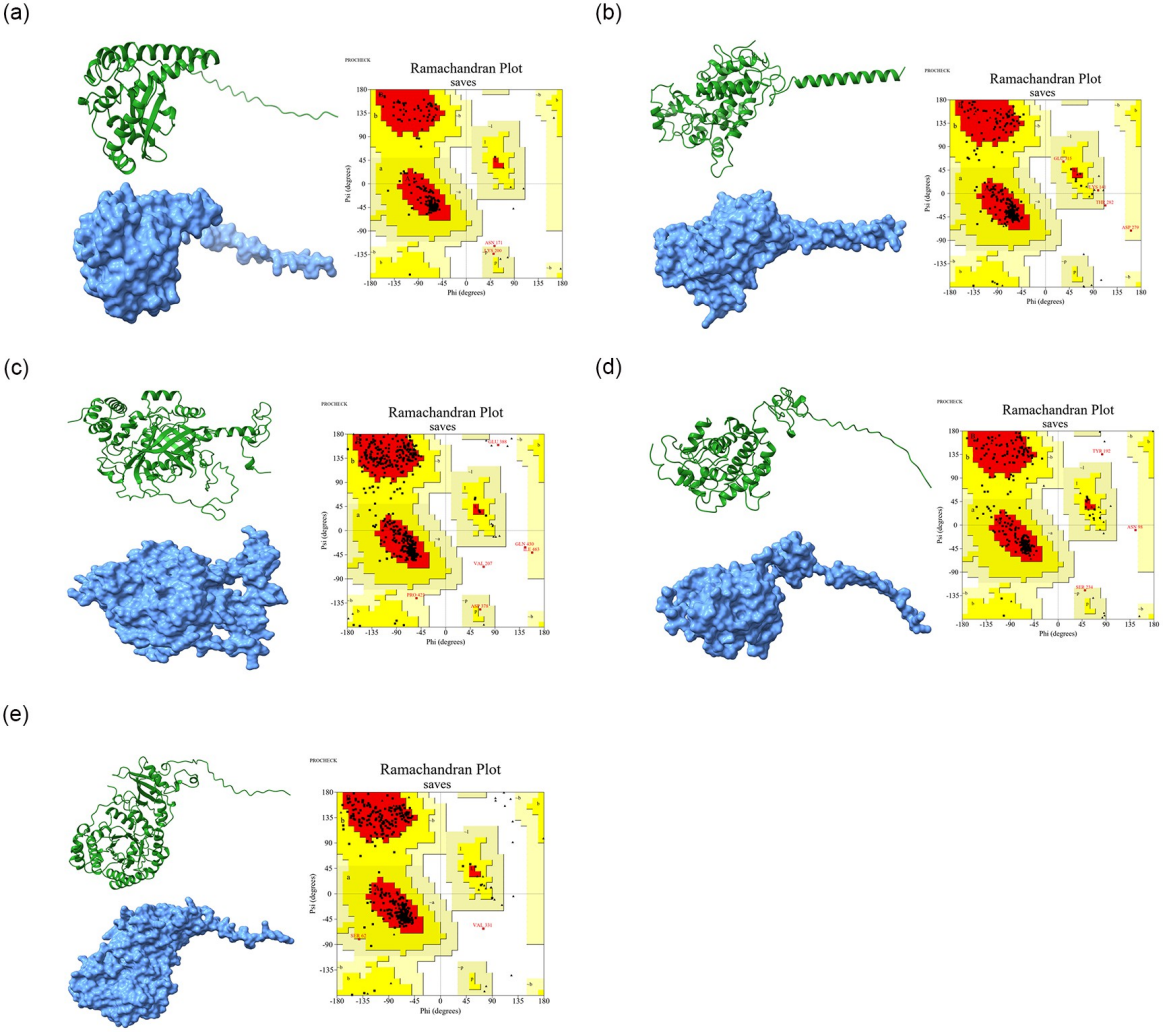
**A**. Three-dimensional ribbon model showed in forest green and surface model showed in cornflower blue color structure of superoxide dismutase enzyme from *Capsicum annuum* predicted through AlphaFold. Verification of superoxide dismutase metabolic enzyme models through Ramachandran plot. Red color showed the most favored region. The yellow color shows the additional allowed region (a, b, l, p). Generally, the allowed region (~a, ~b, ~l, ~p) is highlighted in light yellow color. The Ramachandran plots were generated through precheck tool. **B**. Three-dimensional ribbon model showed in forest green and surface model showed in cornflower blue structure of peroxidase enzyme from *Capsicum annuum* predicted through AlphaFold. Verification of peroxidase metabolic enzyme models through Ramachandran plot. Red color showed the most favored region. The yellow color shows the additional allowed region (a, b, l, p). Generally, the allowed region (~a, ~b, ~l, ~p) is highlighted in light yellow color. The Ramachandran plots were generated through precheck tool. **C**. Three-dimensional ribbon model showed in forest green and surface model showed in corn cornflower blue structure of catalase enzyme from *Capsicum annuum* predicted through AlphaFold. Verification of catalase metabolic enzyme models through Ramachandran plot. Red color showed the most favored region. The yellow color shows the additional allowed region (a, b, l, p). Generally, the allowed region (~a, ~b, ~l, ~p) is highlighted in light yellow color. The Ramachandran plots were generated through precheck tool. **D**. Three-dimensional ribbon model showed in forest green and surface model showed in cornflower blue color structure of chitinase enzyme from *Capsicum annuum* predicted through AlphaFold. Verification of chitinase metabolic enzyme models through Ramachandran plot. Red color showed the most favored region. The yellow color shows the additional allowed region (a, b, l, p). Generally, the allowed region (~a, ~b, ~l, ~p) is highlighted in light yellow color. The Ramachandran plots were generated through precheck tool. **E**. Three-dimensional ribbon model showed in forest green and surface model showed in cornflower blue color structure of ribulose-1,5-bisphosphate carboxylase/oxygenase enzyme from *Capsicum annuum* predicted through AlphaFold. Verification of ribulose-1,5-bisphosphate carboxylase/oxygenase metabolic enzyme models through Ramachandran plot. Red color showed the most favored region. The yellow color shows the additional allowed region (a, b, l, p). Generally, the allowed region (~a, ~b, ~l, ~p) is highlighted in light yellow color. The Ramachandran plots were generated through precheck tool.

**Fig 9:**
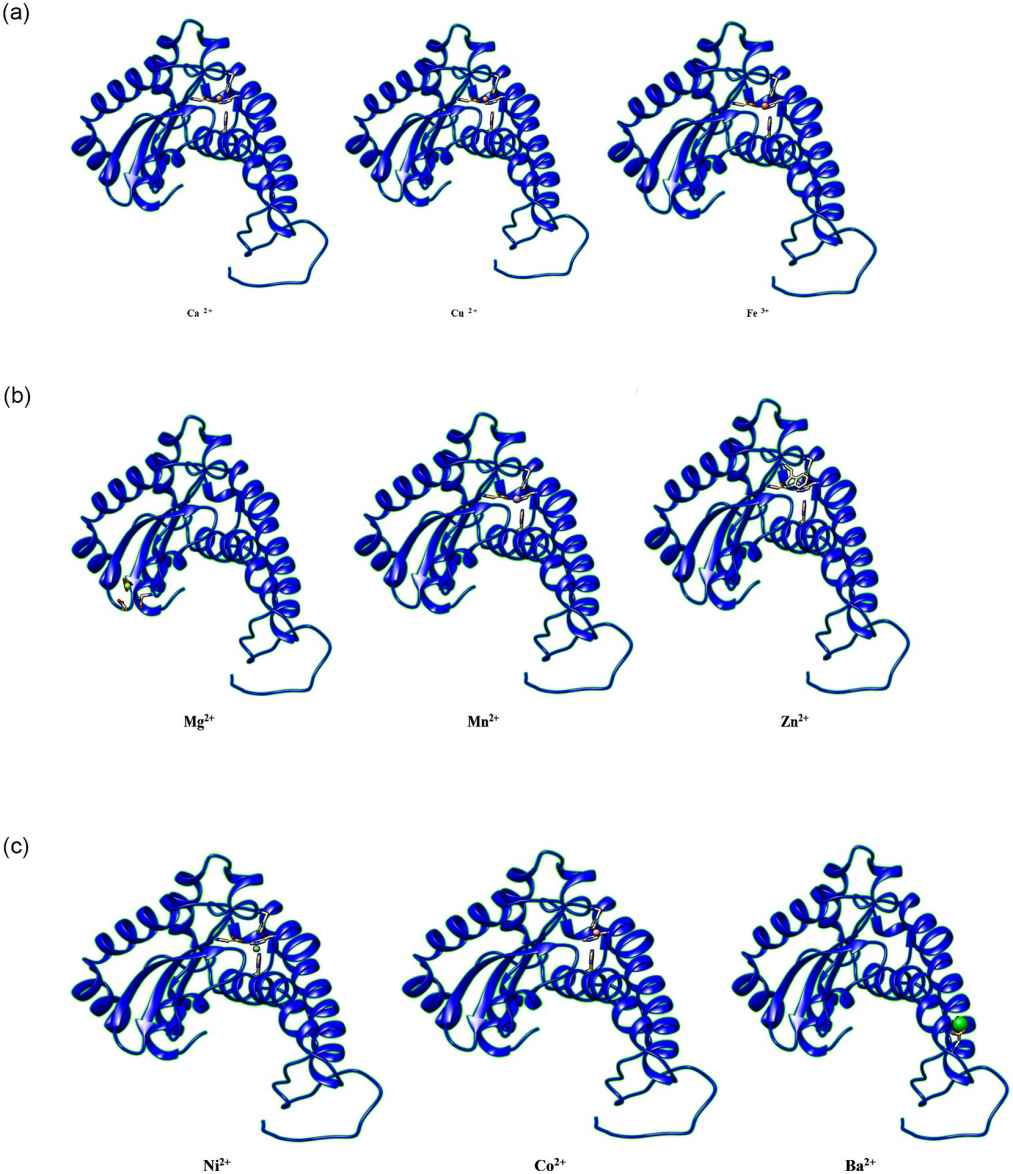
**(a-c)**. Ribbon surface model of superoxide dismutase metabolic enzyme with metals ions. The predicted nutritional ions binding site for Ca^2+^ (52H, 193H), Cu^2+^ (158D, 160E, 181N), Fe^3+^ (52H, 100H, 189D, 193H), Mg^2+^ (158D, 160E, 161L, 181N), Mn^2+^ (52H, 100H, 189D, 193H), Zn^2+^ (189D, 193H), Ni^2+^ (52H, 57H), Co^2+^ (52H, 56H), and Ba^2+^ (79D, 80S, 81P, 82T).

The full-length protein sequences from NCBI database were used to generate 3D model of the superoxide dismutase (NP_001311927.1), peroxidase (AAL35364.1), catalase (NP_001311603.1), chitinase (ACM47315.1), and ribulose-1,5-bisphosphate carboxylase/oxygenase (AID55408.1) metabolic enzymes from *Capsicum annuum*. The 3D model generated through alphaFold was further validated through ERRAT, Verify3D and PROCHECK (Fig 8a–8e). The overall quality of the 3D models was more than 80%, indicating the proper folding of the selected metabolic enzymes (Table 2). The refined 3D models of selected metabolic enzymes were used for docking through the MIB2 server to explore potential metal ions binding sites of selected (Ca2+, Cu2+, Fe3+, Mg2+, Mn2+, Zn2+, Ni2+, Co2+, and Ba2+) plants-based nutrients. The binding sites of metals ions in superoxide dismutase metabolic enzyme were predicted for Ca2+ (52H, 193H), Cu2+ (158D, 160E, 181N), Fe3+(52H, 100H, 189D, 193H), Mg2+(158D, 160E, 161L, 181N), Mn2+ (52H, 100H, 189D, 193H), Zn2+ (189D, 193H), Ni2+ (52H, 57H), Co2+ (52H, 56H), and Ba2+ (79D, 80S, 81P, 82T) (Fig 9a–9c). Similarly, the metal binding sites of (Ca2+, Cu2+, Fe3+, Mg2+, Mn2+, Zn2+, Ni2+, Co2+, and Ba2+) (S2 File) the superoxide dismutase, peroxidase, catalase, chitinase, and ribulose-1,5-bisphosphate carboxylase/oxygenase metabolic enzymes from *Capsicum annuum* were enlisted in Table 3.

**Table 2:** Structural validation of selected enzyme through ERRAT, VERIFY 3D, and PROCHECK.

Amylase Variants	ERRAT	VERIFY 3D	PROCHECK			
	Quality Factor (%)	3D-Score (%)	Most Favoured Region (%)	Additional Allowed Region (%)	Generally Allowed Region (%)	Disallowed Region (%)
Superoxide dismutase	88	97	90.5	8.3	0.6	0.6
Peroxidase	80	80	86.7	11.7	1.2	0.4
Catalase	81	85	88	10	0.5	0.7
Chitinase	84	90	83	14	0.4	0.9
Rubisco	92	87	90.8	8.6	0.3	0.3

**Table 3:** Mineral nutrients binding sites in superoxide dismutase, peroxidase, catalase, chitinase, and ribulose-1,5-bisphosphate carboxylase/oxygenase metabolic enzymes from *Capsicum annuum* perdicted through MIB2.

Metal Ions	Superoxide Dismutase	Peroxidase	Catalase	Chitinase	RuBisCO
Ca^+2^	52H, 193H	75D, 78V, 80G, 82D, 84S	145N, 147D, 149K, 151F, 153D	65D, 71D	203D, 204E, 205N
Cu^+2^	158D, 160E, 181N	72H, 74H, 78V, 93E	199Y, 250H	274E, 277H, 281N	325H, 327H
Fe^3+^	52H, 100H, 189D, 193H	47E, 51Q	146R, 184H, 288D, 425E	240H, 244T	136E, 307H, 310H
Mg^2+^	158D, 160E, 161L, 181N	75D, 79Q, 82D, 84S	236T, 237E, 238E	65D, 71D	203D, 204E
Mn^2+^	52H, 100H, 189D, 193H	153D, 154G	378D, 391D	134Q, 138E, 270N	203D, 205N, 206V
Zn^2+^	189D, 193H	47E, 134R	61E, 108H	25C, 33L, 34C, 39C, 40C	238H, 267H, 292H, 294H
Ni^2+^	52H, 57H	220D, 222S	46H, 50K	280D, 282R	292H, 325H, 327H
Co^2+^	52H, 56H	271E, 274Q	376H, 382N, 385H	274E, 280D	136E, 310H
Ba^2+^	79D, 80S, 81P, 82T	75D, 79Q, 80G, 82D, 85I, 158R	181E, 425E	163S, 166D	115N, 118T

## 4. Discussion

Mineral nutrients have a decisive role in the life of chilli plants as they act as the first line of defense against the invading pathogens by producing various types of enzymes and metabolites [46, 47]. That’s the reason that our study was carried out to induce resistance in chilli plants against Fusarium wilt disease by using macro- and micro- nutrients. In contemporary study, the combination of Krystafeed and Micro Plus (T_3_) demonstrated significant effects in reducing Fusarium wilt disease of chilli under the greenhouse and field conditions. Results of our study are in line with Bashir et al. [48] For the related study, they evaluated Nutritop (Zn, Br, Fe) and Compound (N, P, K) against the Fusarium wilt of chilli, which concluded that the combination of Compound and Nutritop showed significant inhibition effects towards Fusarium wilt. The previous study had shown that the application of macro- and micro- nutrients decreased the percentage of plant mortality caused by different pathogens by regulating different physiological processes (photosynthesis, respiration, transpiration, and defense) by activating different genes [49], which are responsible for the production of various enzymes, such as Rubisco, Transglutaminases (TGases), hexokinase, phosphofructokinase (PFK), acetyl CoA, α-Amylase, Pyruvic kinase, Superoxide dismutase, Peroxidase, Catalase, chitinase, and polyphenol oxidase.

Nitrogen plays an essential role in defensive primary and secondary metabolism [50] by the production of different enzymes and proteins (antimicrobial proteins (AMPs) and pathogen related proteins (PRPs). In response to the infection, incited by pathogens, these PRPs, AMPs, β-glucanase, and chitinase can degrade dextran or chitin present in the cell wall of fungi [51]. Balanced dose of nitrogen increased the activity of defense related enzymes β-1,3-glucanase (PR-2 protein), chitinase (PR3), and chitosanase, which is positively correlated with plant disease resistance [13, 52]. Another study documented the increased expression of PR4 with the increased nitrogen application [53]. N nutrition can also influence defense via amino acid metabolism and hormone production to affect the downstream defense-related gene expression via transcriptional regulation and nitric oxide (NO) production [13]. The above findings clearly indicated that nitrogen fertilizer have positive effects on disease resistance by expressing various pathogenesis-related proteins and enzymes.

Phosphorus application in plants stimulated the production of enzymes, such as acid phosphatases, alkaline phosphatases, and phosphoenolpyruvate carboxylase, which are involved in various metabolic processes. Moreover, P plays a prime role in the plants to perform various functions like coenzymes NAD, NADP, and ATP [54, 55]. Potassium is a key element which is involved in defense mechanism by regulating stomatal regulation, photosynthesis, protein, cell signaling, cellulose, starch formation, hardening of tissues, enzymatic activation, carbohydrate metabolism, and protein synthesis [56, 57]. As potassium regulates more than 60 enzymes in the plant body for the activation of various physiological processes [56], its deficiency directly or indirectly leads towards abnormal functioning and disease development [58].

Manganese involves in the production of phenolic compounds [59], which stimulates the metabolism of carbohydrate α-ketoglutarate (KG), DNA/ RNA formation, and oxidative phosphorylation [60]. As compared to macronutrients, micronutrients are required by plants in small amounts and assist as the major components of prosthetic groups in metalloproteins (catalyze redox processes by electron transfer (Fe, Mn, Cu, Mo) and activators of various enzymatic reactions (coupling enzymes and substrate to form enzyme-substrate complexes (Fe and Zn), which increase enzymatic reactions by altering the molecular configuration of enzymes (Zn). Moreover, the application of zinc is helpful in reducing disease incidence in infected plants through affecting the resistance indirectly by improving structural integrity and permeability of cell membranes [61].

**Fig 10:**
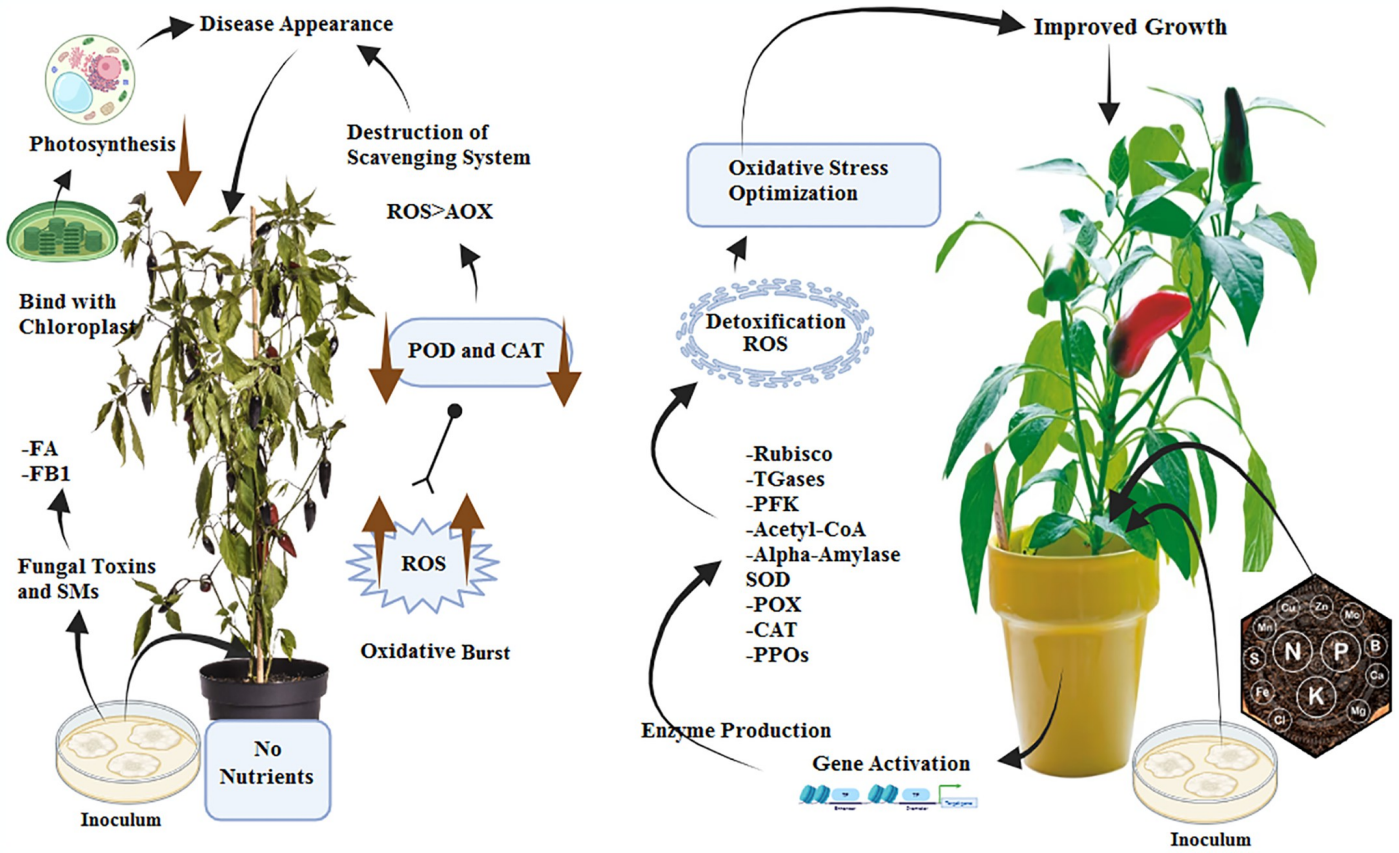
Schematic representation of oxidative stress optimization in chilli plants by the application of plant nutrients.

In our study, the application of macro- and micro- nutrients decreased the disease incidence of Fusarium wilt by increasing superoxide dismutase, peroxidases, catalases, hydrogen peroxide, total phenolic contents, chlorophyll contents (Chlorophyll *a*, chlorophyll *b* and total chlorophyll), tocopherol, thioredoxin reductase, malondialdehyde, ascorbate, phytoalexins, and proline. Results of our study are in line with the previous study of Shoaib and Awan [46]. For their study, they concluded that application of macro- and micro- nutrients (N, P, K, Zn, Mg, B) significantly inhibited the fungal diseases by increasing the activity of components of antioxidant defense system (SOD, POD, CAT, TPC, Chlorophyll contents, PAL, and PPO) (Fig 10).

SOD plays significant roles against various reactive oxygen species (O_2_^-^) and free radicals produced in host plants in response to biotic and abiotic stresses [62, 63]. Moreover, SOD enzyme accumulates H_2_O_2_, which diffuses into cells to act as an excellent antioxidant. This accumulated H_2_O_2_ is used to perform multiple functions in plant defense against invading pathogens. H_2_O_2_ is a very critical component for the synthesis of phytoalexin- defense related compounds, which acts as the retardant at the site of infection by inhibiting pathogen growth, restricts the pathogen spread by triggering programmed cell death (HR), acts as cell to cell signal for the induction of systemic acquired resistance (SAR) [64], and regulates or activates defense related genes [65]. However, the excessive amount of H_2_O_2_ is lethal to plants, which can be neutralized by two enzymes named POD and CAT. These two enzymes prevent the accumulation of H_2_O_2_ in the cells and converts it into water and oxygen [66].

Total phenolic contents (TPC) protect the major tissues of plants from the lethal effects of ROS and help plants to evade pathogenic infections [67] by minimizing the effects to enzymes, which degrade plant cell wall, inhibit the entry of pathogen secreted enzymes and toxins into plant cells, and stops the flow of nutrients towards pathogens [68]. Proline plays its significant role in plant defense activation by contributing as signaling molecule, antioxidative defense molecule, and metal chelator [69]. Catabolism and biosynthesis of proline produce pyrroline-5-carboxylate (P_5_C) which plays a significant role in both non-host and *R-gene* mediated resistance against invading pathogens [70].

Tocopherols are lipid soluble antioxidant molecules, present in plastids and thylakoid membrane, which are responsible for conforming resistance in plants by reducing ROS level in photosynthetic machinery and protect the thylakoid membrane from lipid peroxidation against various biotic stresses [71, 72]. Thioredoxin reductase (TrxR) plays a crucial role in plant defense by maintaining cellular redox homeostasis, regulating gene expression, modulating protein function, and detoxifying reactive oxygen and nitrogen species [73]. Lipid peroxidation is a process which initiates under oxidative stress conditions when reactive oxygen species (ROS) attack polyunsaturated fatty acids in cell membranes, which increase the production of malondialdehyde (MDA). While MDA itself does not have a direct role in plant defense, its accumulation is often used as a marker for oxidative damage and stress in plants [74]. Phytoalexins are low-molecular-weight antimicrobial compounds produced by plants in response to pathogen attack or other stresses [75]. Capsidiol accumulates at the site of infection and acts as a defense molecule by inhibiting the growth and proliferation of the invading pathogens. It directly interferes with pathogen metabolism, disrupts cellular processes, and hampers pathogen survival and reproduction. Our study is supported by the above findings, which clearly justifies the role of various biochemical constituents in plant defense and enhancement of these enzymes through the application of macro- and micro- nutrients, leading towards the activation of defense related responses.

## 5. Conclusion

This study investigated the role of plant nutrients against Fusarium wilt disease of chilli. Results of our study confirmed that the application of macro- and micro- nutrient significantly reduced the incidence of Fusarium wilt in chilli plants. Moreover, the application of T_3_ (Krystafeed 400 and Microplus 40 g/100ft^2^) significantly improved the biochemical constituents (SOD, POD, CAT, H_2_O_2_, TrxR, MDA, Ascorbate, Chlorophyll contents, Phenolic contents, and Capsidiol) of chilli plant (S1 File). In conclusion, the application of plant nutrients can be a viable and healthy approach for the management of Fusarium wilt of chilli and potentially other pathogen controls.

## Supporting information

S1 File Raw data of manuscript which was subjected to statistical analysis. (XLSX)

S2 File Supplementary data of enzymes and metal ions. (ZIP)
