# Supplementary material for: Computational and experimental approaches to explore defense related enzymes conferring resistance in Fusarium infected chilli plants by regulating plant metabolism through nutritional products
Source: PLoS One. 2025 Jan 14;20(1):e0309738. doi: 10.1371/journal.pone.0309738 (PMC11731765; doi:10.1371/journal.pone.0309738)
Supplement: S2 File — (ZIP) [file pone.0309738.s002.zip › USMAN PAPER/Enzymes and Metal Ions Data/SAVES RESULTS.docx]

Structural validation of selected enzyme through ERRAT, VERIFY 3D, and PROCHECK

| **Amylase Variants** | **ERRAT** | **VERIFY 3D** | **PROCHECK** | | | |
| --- | --- | --- | --- | --- | --- | --- |
|  | Quality Factor  (%) | 3D-Score  (%) | Most Favoured Region  (%) | Additional Allowed Region (%) | Generally Allowed Region (%) | Disallowed  Region  (%) |
| Superoxide dismutase | 88 | 97 | 90.5 | 8.3 | 0.6 | 0.6 |
| Peroxidase | 80 | 80 | 86.7 | 11.7 | 1.2 | 0.4 |
| Catalase | 81 | 85 | 88 | 10 | 0.5 | 0.7 |
| Chitinase | 84 | 90 | 83 | 14 | 0.4 | 0.9 |
| Rubisco | 92 | 87 | 90.8 | 8.6 | 0.3 | 0.3 |
|  |  |  |  |  |  |  |
