# Supplementary material for: Computational and experimental approaches to explore defense related enzymes conferring resistance in Fusarium infected chilli plants by regulating plant metabolism through nutritional products
Source: PLoS One. 2025 Jan 14;20(1):e0309738. doi: 10.1371/journal.pone.0309738 (PMC11731765; doi:10.1371/journal.pone.0309738)
Supplement: S2 File — (ZIP) [file pone.0309738.s002.zip › USMAN PAPER/Enzymes and Metal Ions Data/ENZYME SEQUENCES.docx]

**ribulose-1,5-bisphosphate carboxylase/oxygenase large subunit, partial (chloroplast) [Capsicum annuum]**

GenBank: AID55408.1

[GenPept](https://www.ncbi.nlm.nih.gov/protein/AID55408.1?report=genpept) [Identical Proteins](https://www.ncbi.nlm.nih.gov/ipg/AID55408.1) [Graphics](https://www.ncbi.nlm.nih.gov/protein/AID55408.1?report=graph)

>AID55408.1 ribulose-1,5-bisphosphate carboxylase/oxygenase large subunit, partial (chloroplast) [Capsicum annuum]

MSPQTETKASVGFKAGVKEYKLTYYTPEYQTKDTDILAAFRVTPQPGVPPEEAGAAVAAESSTGTWTTVW

TDGLTSLDRYKGRCYRIERVVGEKDQYIAYVAYPLDLFEEGSVTNMFTSIVGNVFGFKALRALRLEDLRV

PTAYIKTFQGPPHGIQVERDKLNKYGRPLLGCTIKPKLGLSAKNYGRAVYECLRGGLDFTKDDENVNSQP

FMRWRDRFLFCAEALFKAQTETGEIKGHYLNATAGTCEEMMKRAVFARELGAPIVMHDYLTGGFTANTSL

AHYCRDNGLLLHIHRAMHAVIDRQKNHGMHFRVLAKALRMSGGDHIHAGTVVGKLEGERDITLGFVDLLR

DDFVEQDRSRGIYFTQDWVSLPGVLPVASGGIHVWHMPALTEIFGDDSVLQFGGGTLGHPWGNAPGAVAN

# hexokinase family protein [Capsicum annuum]

GenBank: KAF3636144.1

[GenPept](https://www.ncbi.nlm.nih.gov/protein/KAF3636144.1?report=genpept) [Identical Proteins](https://www.ncbi.nlm.nih.gov/ipg/KAF3636144.1) [Graphics](https://www.ncbi.nlm.nih.gov/protein/KAF3636144.1?report=graph)

>KAF3636144.1 hexokinase family protein [Capsicum annuum]

MAMCSIYKYGSKAQGVAFLAFGSISFLVFVYVAIVSKLLPPFDNPILAAIQNDRRSRVQALETVSGRNAS

LQHNIVYWHNPLVIVDILLLSGSINASHPRCCNIFPLAQYEDLQACITPMRYGAFASSPEDFYDMVRFLF

RSEFFT

# ATP-dependent 6-phosphofructokinase 6 isoform X1 [Capsicum annuum]

NCBI Reference Sequence: XP_016537634.1

[GenPept](https://www.ncbi.nlm.nih.gov/protein/XP_016537634.1?report=genpept) [Identical Proteins](https://www.ncbi.nlm.nih.gov/ipg/XP_016537634.1) [Graphics](https://www.ncbi.nlm.nih.gov/protein/XP_016537634.1?report=graph)

>XP_016537634.1 ATP-dependent 6-phosphofructokinase 6 isoform X1 [Capsicum annuum]

MGTESKYQMKVVTGDYGYVLEDVPHLTDYIPDLPTYDNPLQSNPAYSVVKQYFVHMDDTVPQKIVVHKDS

QRGVHFRRAGPRQKVYFSSDDVRACIVTCGGLCPGLNTVIREIVHNLDYIYGVDKVLGIEGGYRGFYAKN

TINLTPKLVNDIHKRGGTILGTSRGGHDTTKIVDSIQDHGINQVYIIGGDGTQKGAAFIYEEIRRRGLKV

VVAGIPKTIDNDIPVIDKSFGFDTAVEEAQRAINAAHVEAQSAENGIGLVKLMGRYSGFIAMYATLASRD

VDCCLIPESPFFLEGSGGLFEFVKKRLREEGHMVIVIAEGAGQELLAAENSHARSEQDASGNKLLQDVGL

WVSHKIRDHFAKKLKMPITLKYIDPTYMIRAVPSNASDNVYCTILAQSCVHGAMAGYTGFTSGVVNGRQT

YIPFNRITEKQNKVVITDRMWARLLSSTSQPSFLSTSDIVQLQKRQHSQTQLLGGDNNESEITGQQNTST

A

# superoxide dismutase [Mn], mitochondrial [Capsicum annuum]

NCBI Reference Sequence: NP_001311927.1

[GenPept](https://www.ncbi.nlm.nih.gov/protein/NP_001311927.1?report=genpept) [Identical Proteins](https://www.ncbi.nlm.nih.gov/ipg/NP_001311927.1) [Graphics](https://www.ncbi.nlm.nih.gov/protein/NP_001311927.1?report=graph)

>NP_001311927.1 superoxide dismutase [Mn], mitochondrial [Capsicum annuum]

MALRNLMTKKPFAGILTFRQQLRCVQTFSLPDLSYDYGALEPAISGEIMQLHHQKHHQTYITNYNNALQQ

LHDAINKGDSPTVAKLQGAIKFNGGGHINHSVFWKNLAPTREGGGEPPKGSLGSAIDTNFGSLEAVIQKM

NAEGAALQGSGWVWLGLDKELKRLVIETTANQDPLVIKGPNLVPLLGIDVWEHAYYLQYKNVKPDYLKNI

WKVINWKYAAEVYEKECP

# peroxidase [Capsicum annuum]

GenBank: AAL35364.1

[GenPept](https://www.ncbi.nlm.nih.gov/protein/AAL35364.1?report=genpept) [Identical Proteins](https://www.ncbi.nlm.nih.gov/ipg/AAL35364.1) [Graphics](https://www.ncbi.nlm.nih.gov/protein/AAL35364.1?report=graph)

>AAL35364.1 peroxidase [Capsicum annuum]

MEYYYNYNSINKMVSIIFILVLAIDLTMVLGQGTRVGFYSSTCPRAESIVQSTVRSHFQSDPTVAPGLLT

MHFHDCFVQGCDASILISGSGTERTAPPNSLLRGYEVIDDAKQQIEAICPGVVSCADILALAARDSVLVT

KGLTWSVPTGRRDGLVSRASDTSDLPGFTESVDSQKQKFSAKGLNTQDLVTLVGGHTIGTSACQFFSYRL

YNFNSTGGPDPSIDASFLPTLRGLCPQNGDGSKRVALDTGSVNNFDTSYFSNLRNGRGILESDQKLWTDD

STKVFIQRYLGLRGFLGLRFGVEFGRSMVKMSNIEVKTGTNGEIRKVCSAIN

# catalase [Capsicum annuum]

NCBI Reference Sequence: NP_001311603.1

[GenPept](https://www.ncbi.nlm.nih.gov/protein/NP_001311603.1?report=genpept) [Identical Proteins](https://www.ncbi.nlm.nih.gov/ipg/NP_001311603.1) [Graphics](https://www.ncbi.nlm.nih.gov/protein/NP_001311603.1?report=graph)

>NP_001311603.1 catalase [Capsicum annuum]

MDLSKYRPSSAYDSPFLTTNAGGPVYNNVSSLTVGPRGPVLLEDYHLIEKLATFDRERIPERVVHARGAS

AKGFFEVTHDISHLTCADFLRAPGVQTPVICRFSTVVHTRGSPESIRDILGFAVKFYTREGNFDLVGNNV

PVFFNRDTKSFPDTIRALKPNPKSHIQENWRILDFFSFLPESLHTFAFFYDDVCLPTDYRHMEGFGVHAY

QLINKAGKAHYVKFHWKPTCGVKSMTEEEAIRVGGTNHSHATKDLYDSIAAGNYPEWKLFIQIMNPEDVD

KFDFDPLDVTKTWPEDILPLMPVGRLVLNRNIDNFFAENEQLAFNPGHIVPGVYYSEDKLLQTRIFAYAD

TQRHRIGPNYMQLPVNAPKCAHHNNHRDGAMNFMHRDEEVDYLPSRFDPCRPAEQYPIPSCVLTGRREKC

VIPKENNFKQAGERYRSWAPDRQDRYINKWVESLSDPRVTHEIRSIWISYLSQADKSCGQKVASRLTVKP

TM

# chitinase [Capsicum annuum]

GenBank: ACM47315.1

[GenPept](https://www.ncbi.nlm.nih.gov/protein/ACM47315.1?report=genpept) [Identical Proteins](https://www.ncbi.nlm.nih.gov/ipg/ACM47315.1) [Graphics](https://www.ncbi.nlm.nih.gov/protein/ACM47315.1?report=graph)

>ACM47315.1 chitinase [Capsicum annuum]

MRLSEFSFFSLLFAVLLLAVSAEQCGSQAGGALCAAGLCCSKFGWCGNTNDYCGAGNCQSQCPGDSGPTG

DLGSIISNSMFDEMLKHRNDNACQGKNNFYSYNAFITAAKSFPGFGTTGDTAVRKREIAAFFAQTSHETT

GGWPTAPDGPYAWGYCFLREQGSPGDYCSPSGQWPCAPGRKYFGRGPIQISYNYNYGPCGRAIGVDLLNN

PDLVATDSVISFKSAIWFWMTPQSPKPSCHDVITGRWQPSSADRAANRLPGFGVITNIINGGLECGHGND

NRVQDRIGFYRRYCGILGVSPGDNLDCGNQRSFGNGLLVDIM
