# Supplementary material for: Computational and experimental approaches to explore defense related enzymes conferring resistance in Fusarium infected chilli plants by regulating plant metabolism through nutritional products
Source: PLoS One. 2025 Jan 14;20(1):e0309738. doi: 10.1371/journal.pone.0309738 (PMC11731765; doi:10.1371/journal.pone.0309738)
Supplement: S2 File — (ZIP) [file pone.0309738.s002.zip › USMAN PAPER/Enzymes and Metal Ions Data/saves_01 (1).pdf]

PROCHECK

Ramachandran Plot

saves

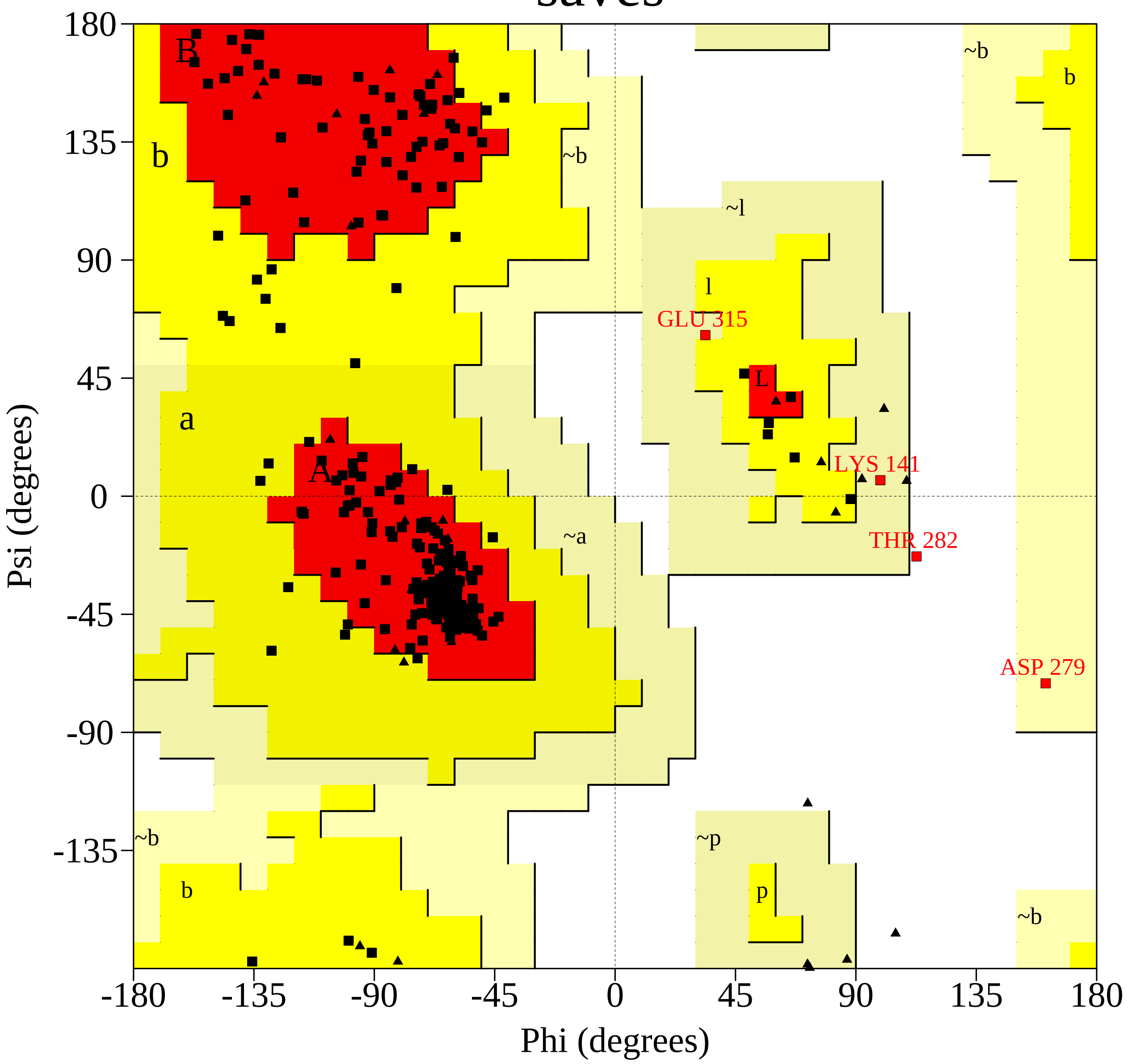

Plot statistics

Residues in most favoured regions [A,B,L]	222	86.7%
Residues in additional allowed regions [a,b,l,p]	30	11.7%
Residues in generously allowed regions [~a,~b,~l,~p]	3	1.2%
Residues in disallowed regions	1	0.4%
	----	------
Number of non-glycine and non-proline residues	256	100.0%
Number of end-residues (excl. Gly and Pro)	1	
Number of glycine residues (shown as triangles)	31	
Number of proline residues	12	

Total number of residues	300	

Based on an analysis of 118 structures of resolution of at least 2.0 Angstroms and R-factor no greater than 20%, a good quality model would be expected to have over 90% in the most favoured regions.
