# Supplementary figures and images for: Computational and experimental approaches to explore defense related enzymes conferring resistance in Fusarium infected chilli plants by regulating plant metabolism through nutritional products
Source: PLoS One. 2025 Jan 14;20(1):e0309738. doi: 10.1371/journal.pone.0309738 (PMC11731765; doi:10.1371/journal.pone.0309738)

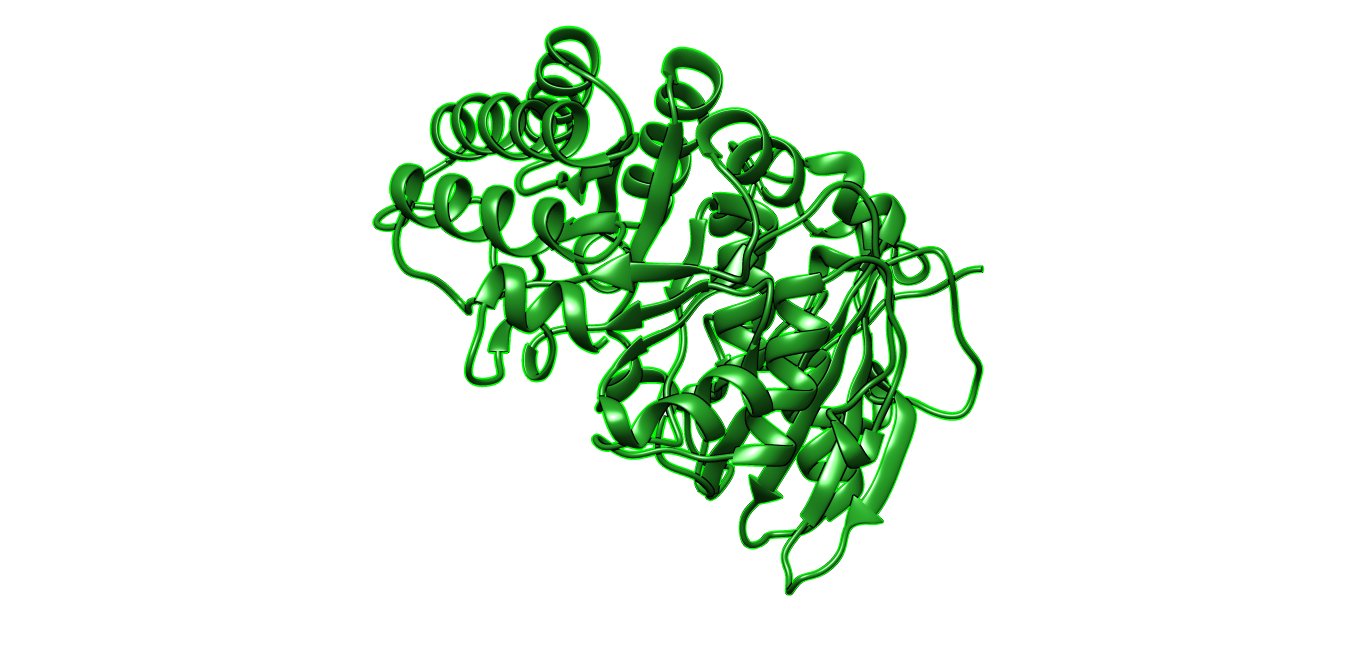

Supplement: S2 File — (ZIP) [file pone.0309738.s002.zip › USMAN PAPER/Enzymes and Metal Ions Data/RIBISCO-R_1.jpg]

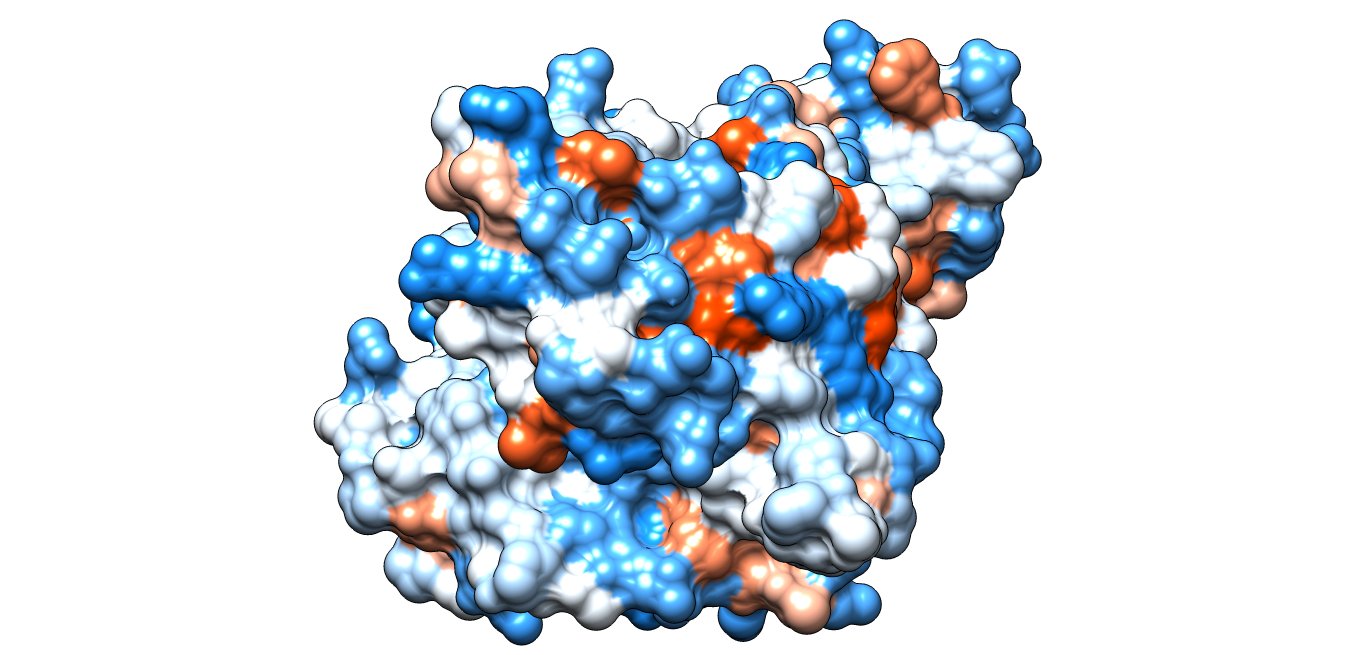

Supplement: S2 File — (ZIP) [file pone.0309738.s002.zip › USMAN PAPER/Enzymes and Metal Ions Data/CHITINASE-S_1.jpg]

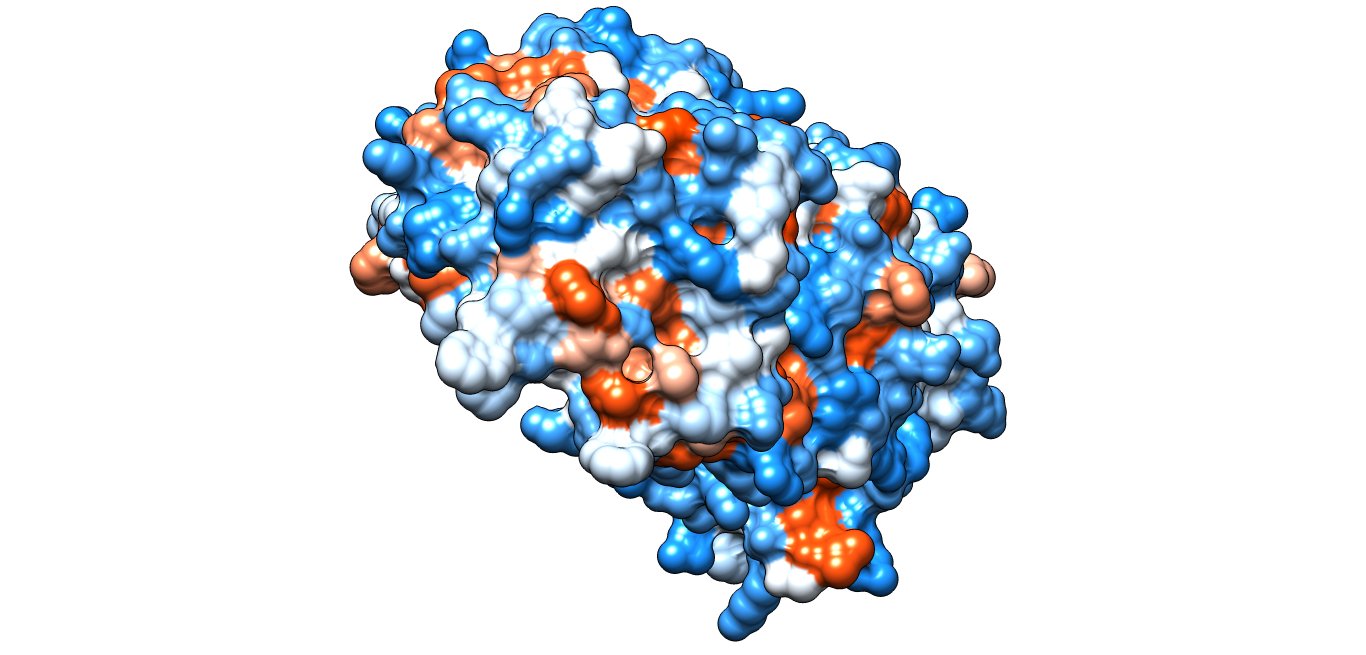

Supplement: S2 File — (ZIP) [file pone.0309738.s002.zip › USMAN PAPER/Enzymes and Metal Ions Data/RIBISCO-S_1.jpg]

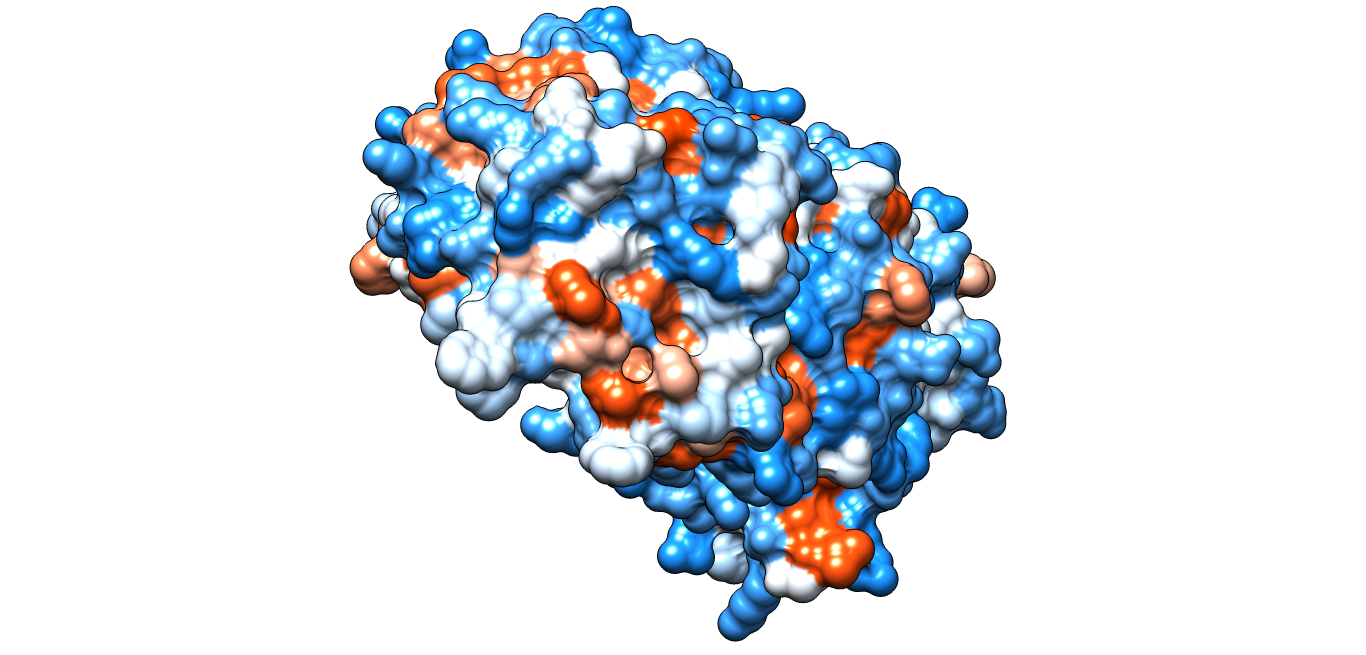

Supplement: S2 File — (ZIP) [file pone.0309738.s002.zip › USMAN PAPER/Enzymes and Metal Ions Data/RIBISCO-S.tif]

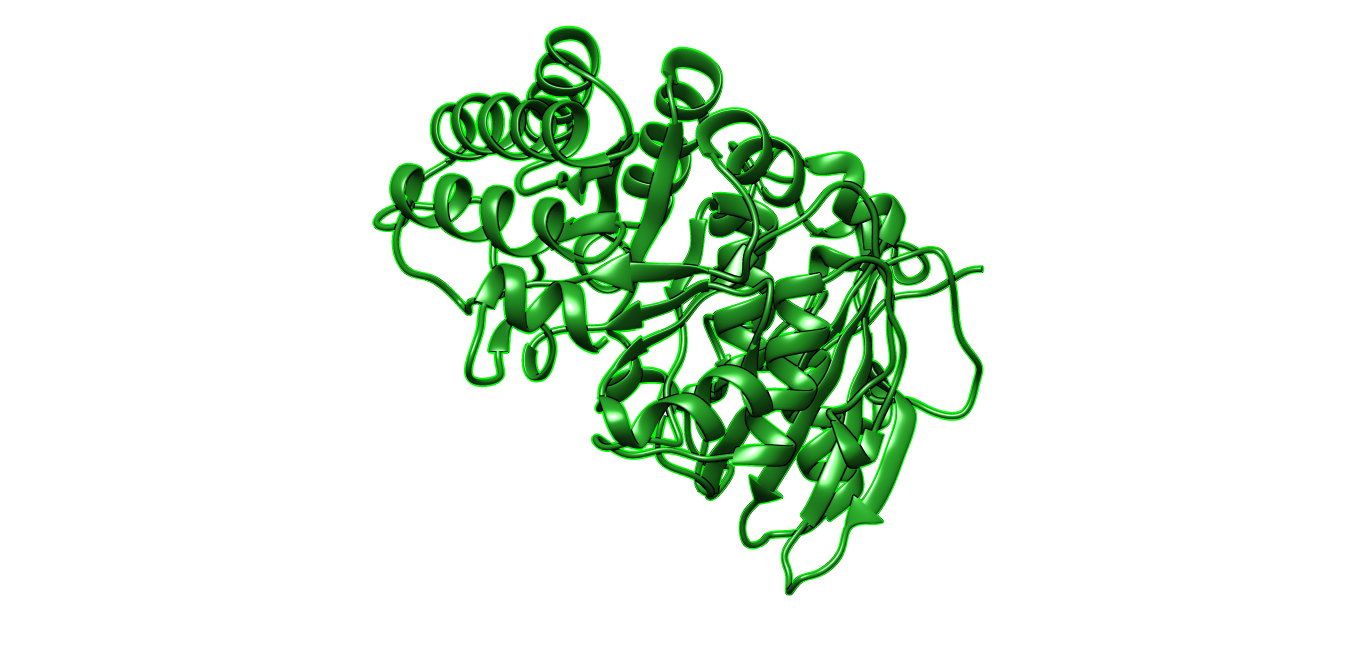

Supplement: S2 File — (ZIP) [file pone.0309738.s002.zip › USMAN PAPER/Enzymes and Metal Ions Data/RIBISCO-R.tif]

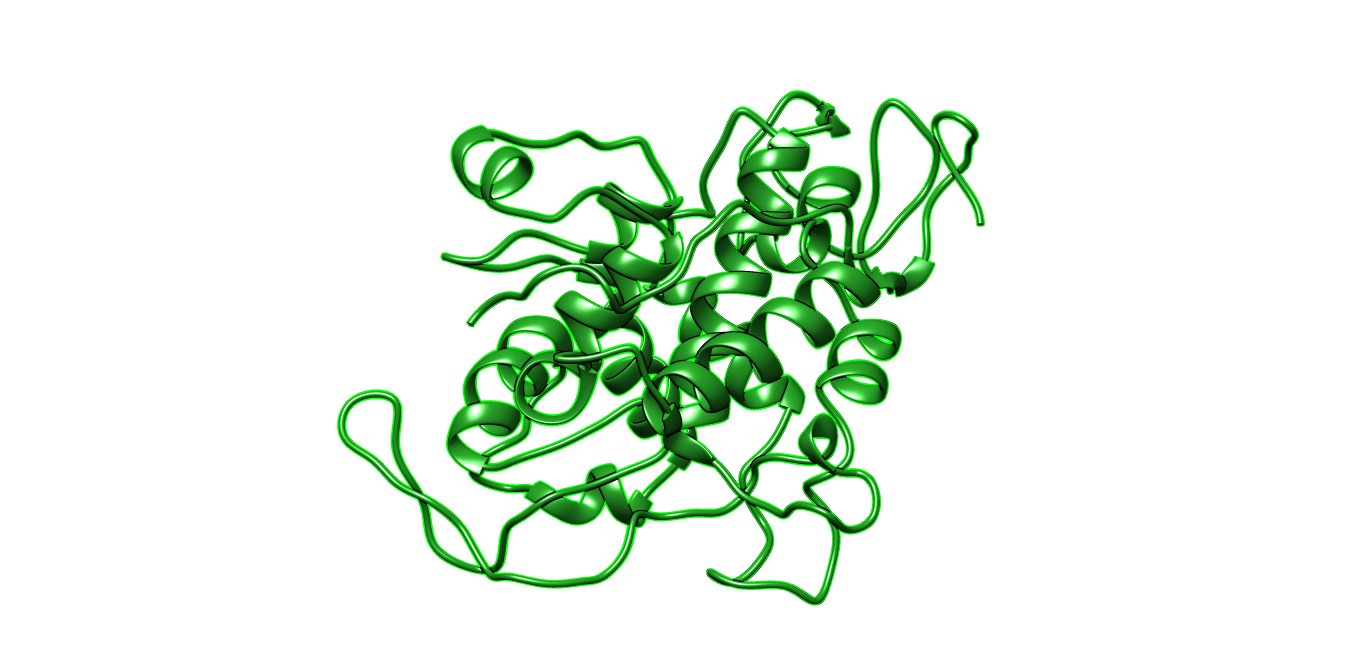

Supplement: S2 File — (ZIP) [file pone.0309738.s002.zip › USMAN PAPER/Enzymes and Metal Ions Data/CHITINASE-R_1.jpg]

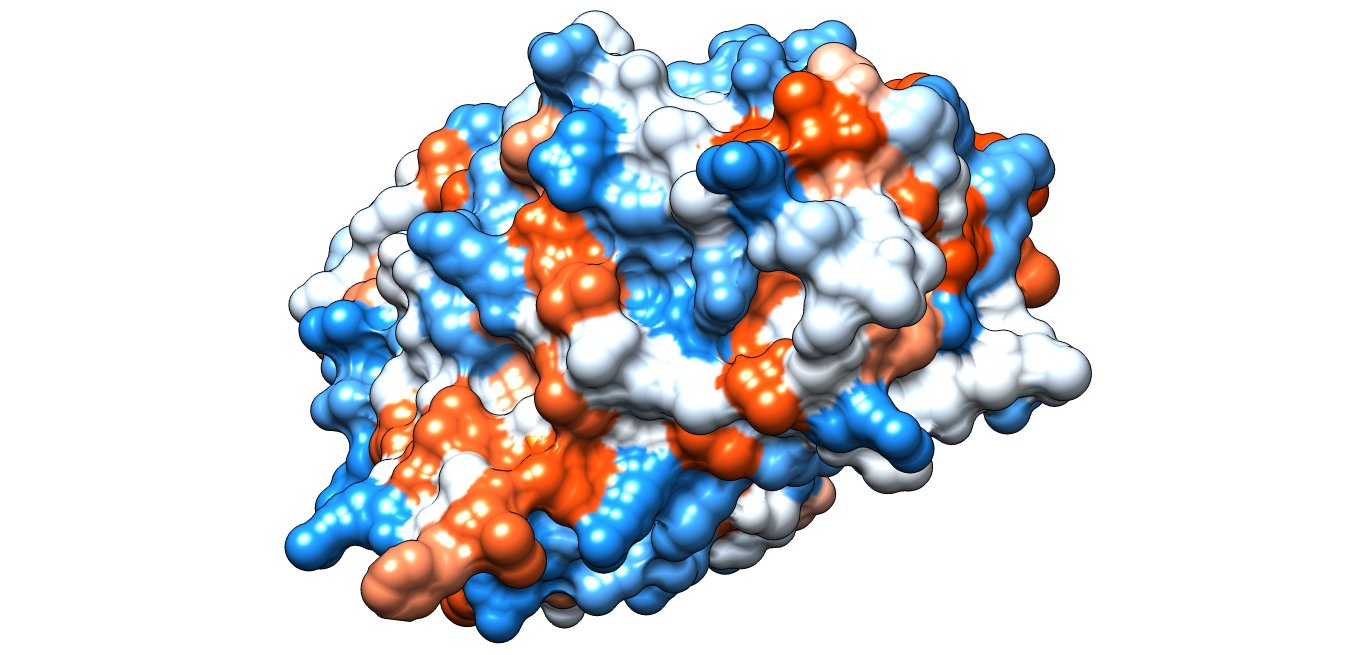

Supplement: S2 File — (ZIP) [file pone.0309738.s002.zip › USMAN PAPER/Enzymes and Metal Ions Data/PEROXIDASE-S_1.jpg]

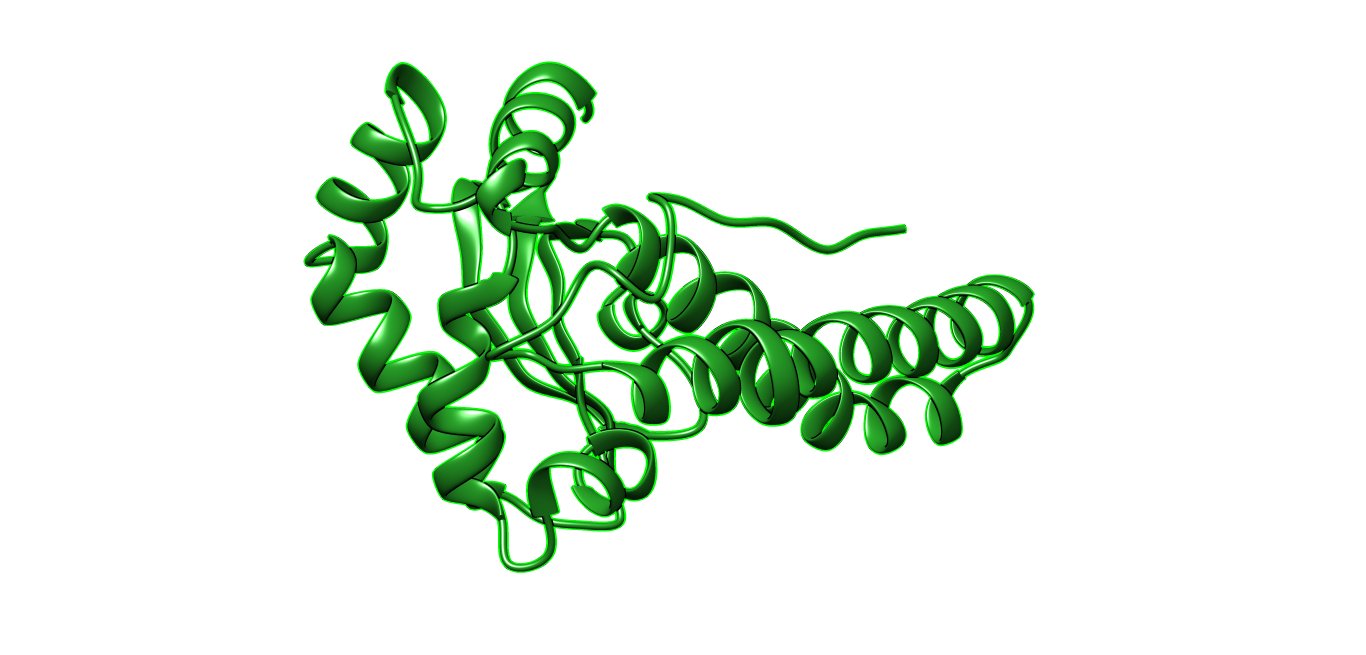

Supplement: S2 File — (ZIP) [file pone.0309738.s002.zip › USMAN PAPER/Enzymes and Metal Ions Data/SOD-R_1.jpg]

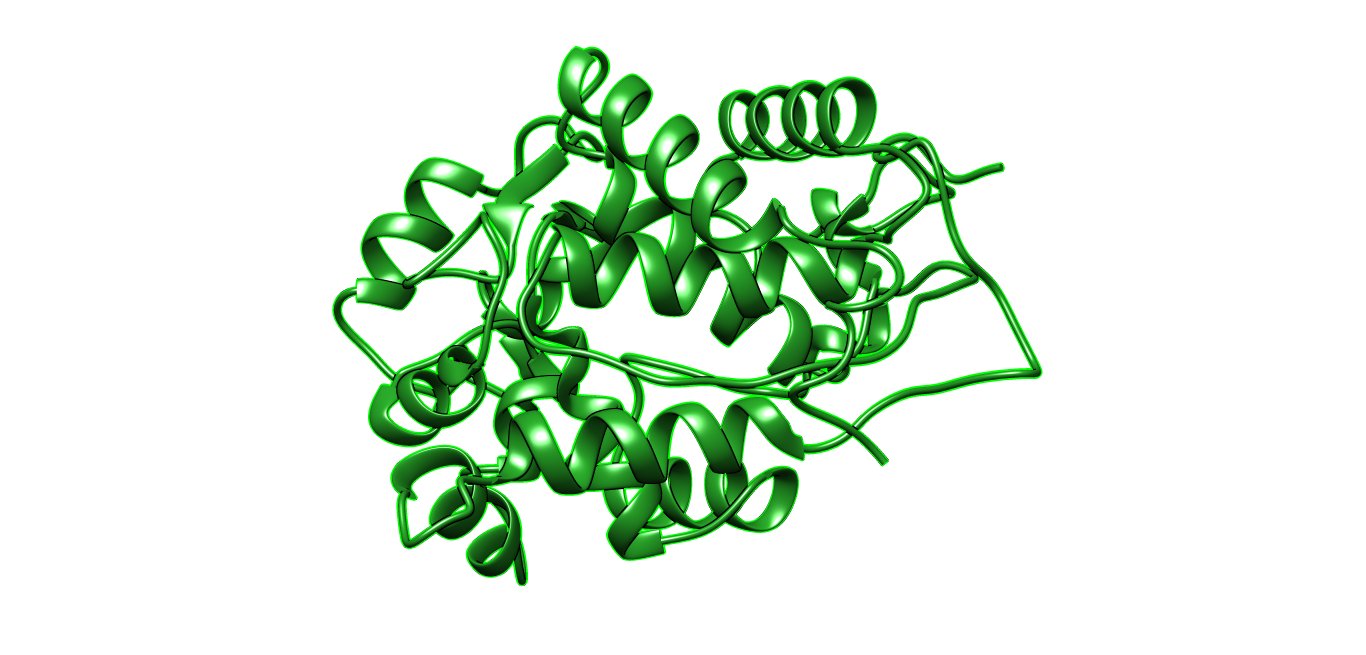

Supplement: S2 File — (ZIP) [file pone.0309738.s002.zip › USMAN PAPER/Enzymes and Metal Ions Data/PEROXIDASE-R_1.jpg]

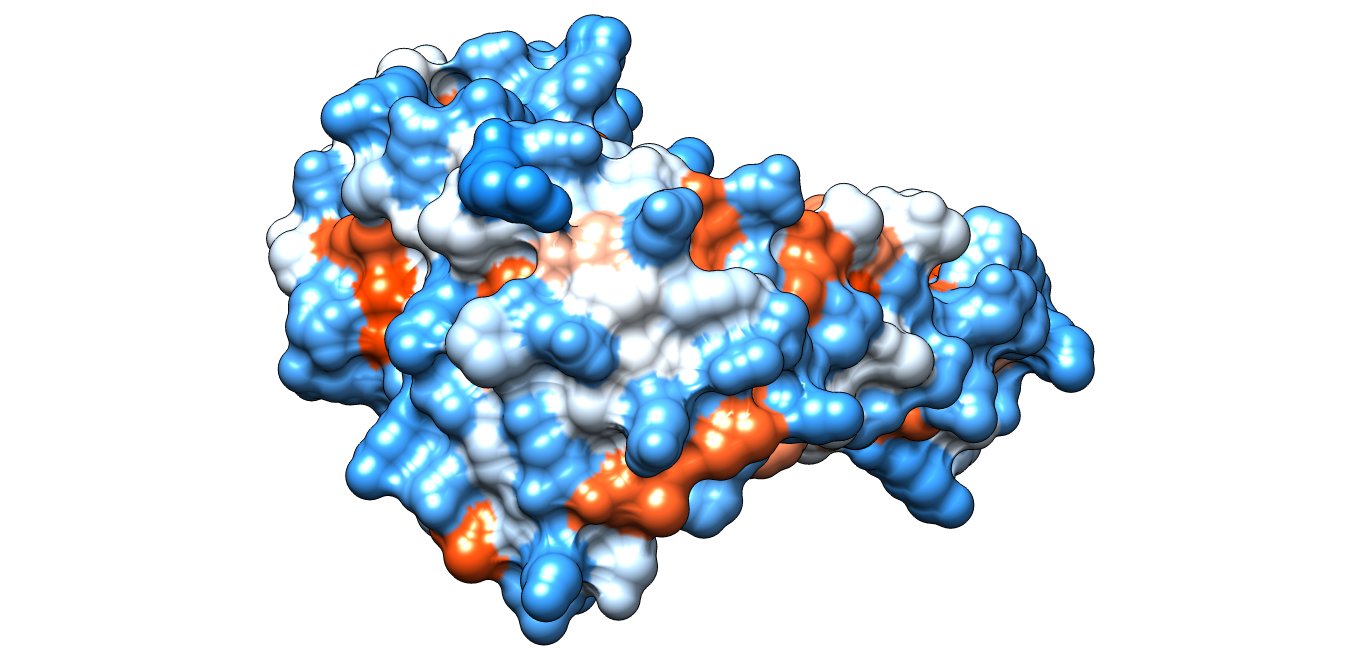

Supplement: S2 File — (ZIP) [file pone.0309738.s002.zip › USMAN PAPER/Enzymes and Metal Ions Data/SOD-S_1.jpg]

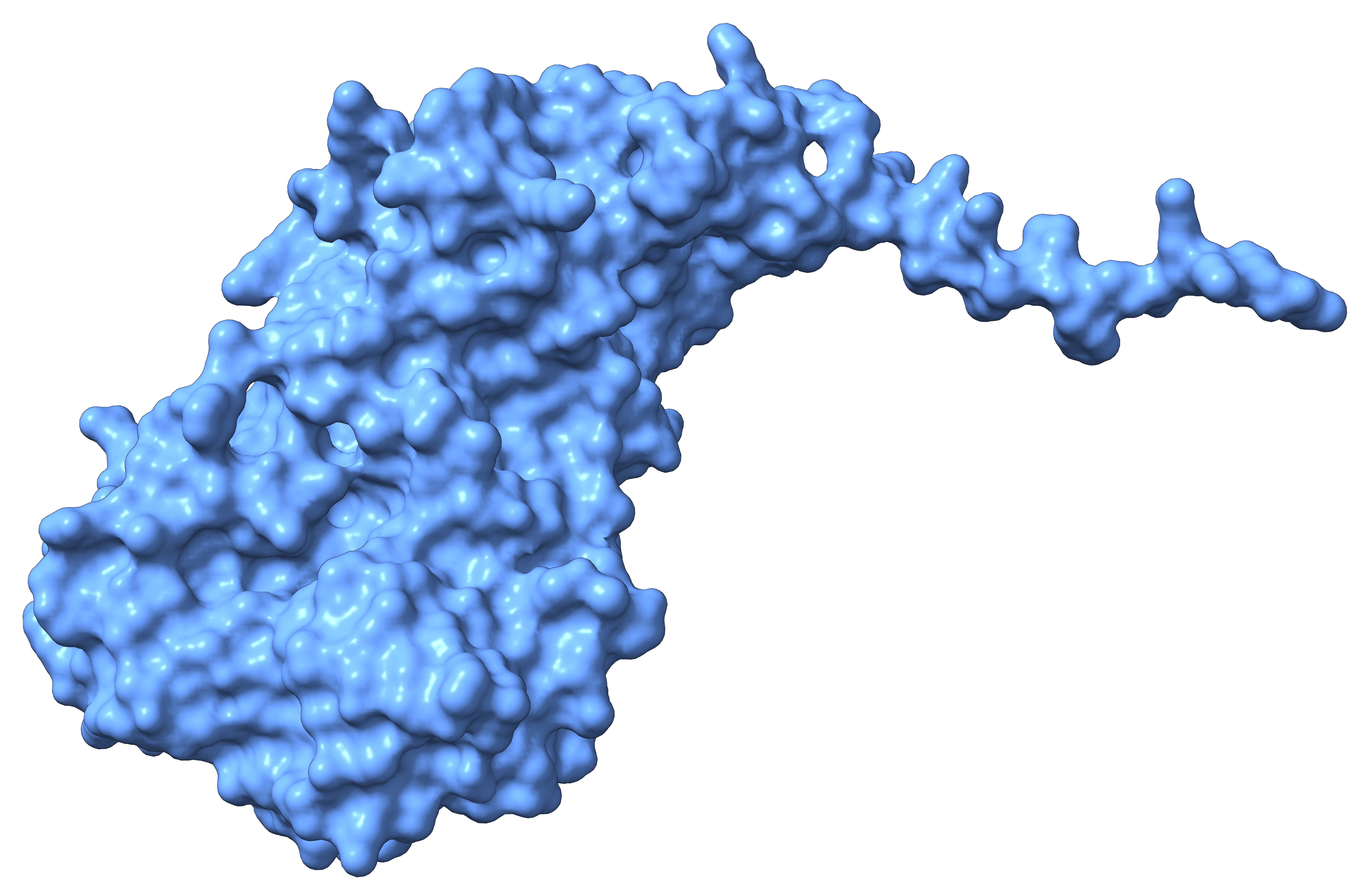

Supplement: S2 File — (ZIP) [file pone.0309738.s002.zip › USMAN PAPER/Structure of Enzymes/RIBULOSE-S.jpg]

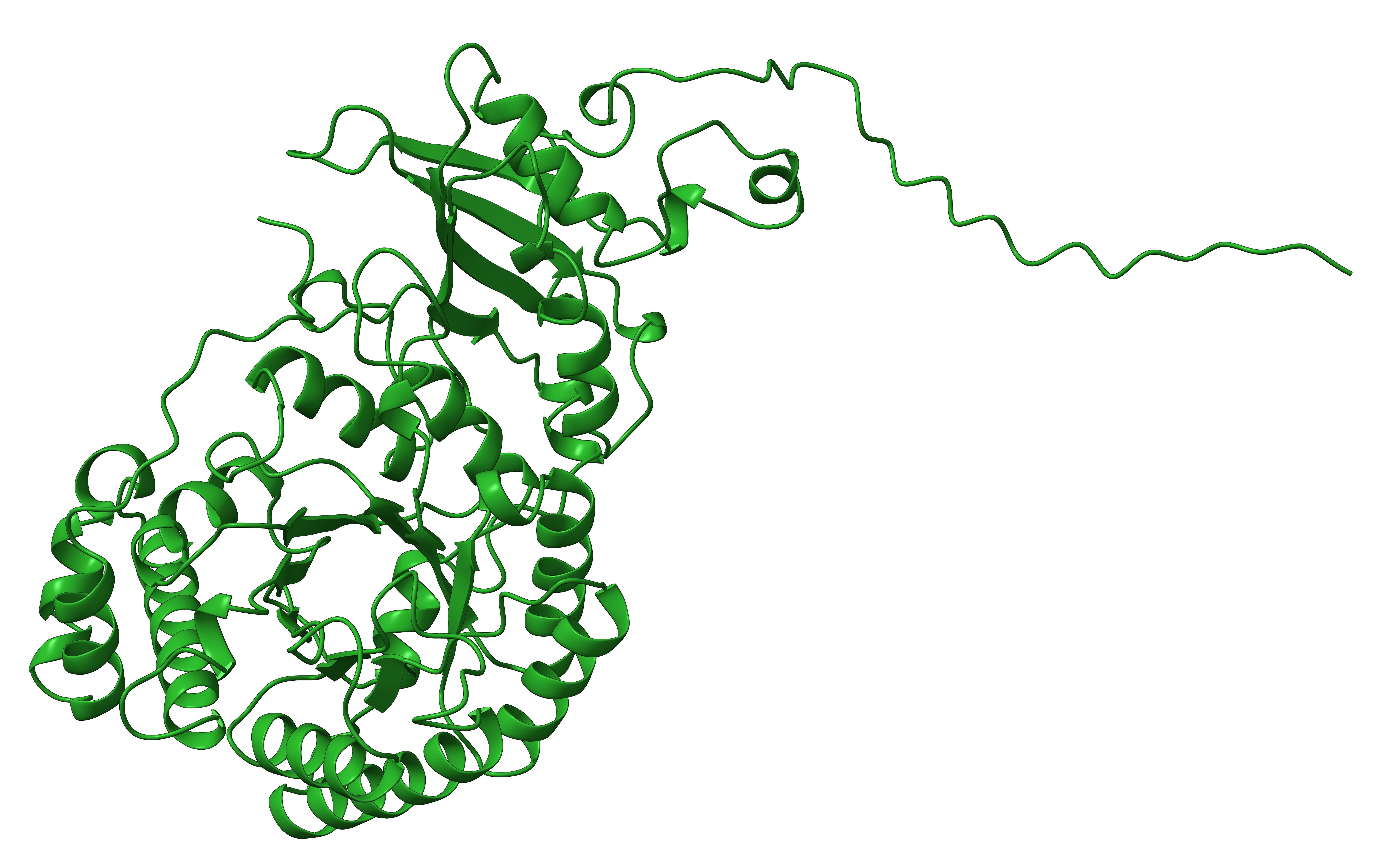

Supplement: S2 File — (ZIP) [file pone.0309738.s002.zip › USMAN PAPER/Structure of Enzymes/RIBULOSE-R.jpg]

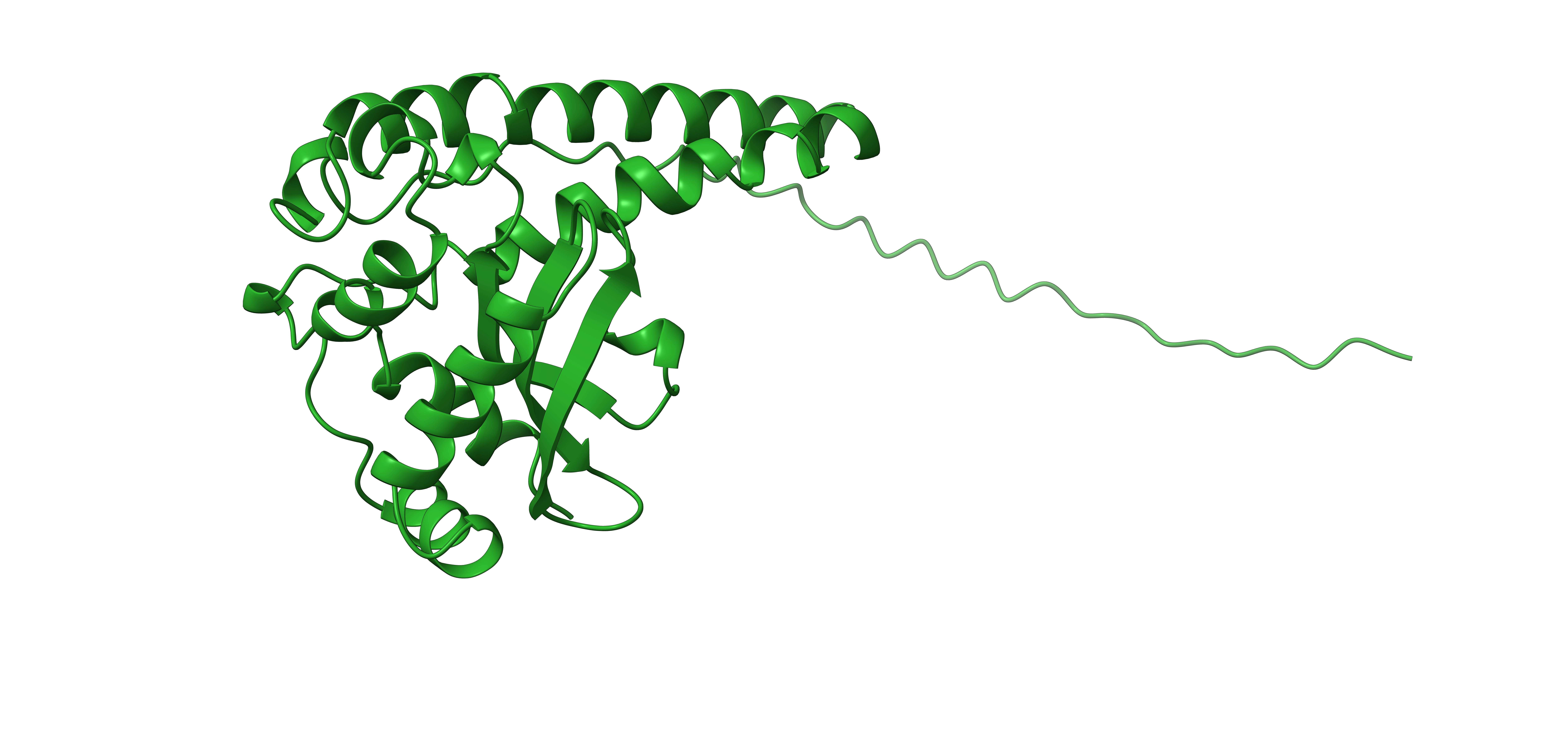

Supplement: S2 File — (ZIP) [file pone.0309738.s002.zip › USMAN PAPER/Structure of Enzymes/SOD-R.jpg]

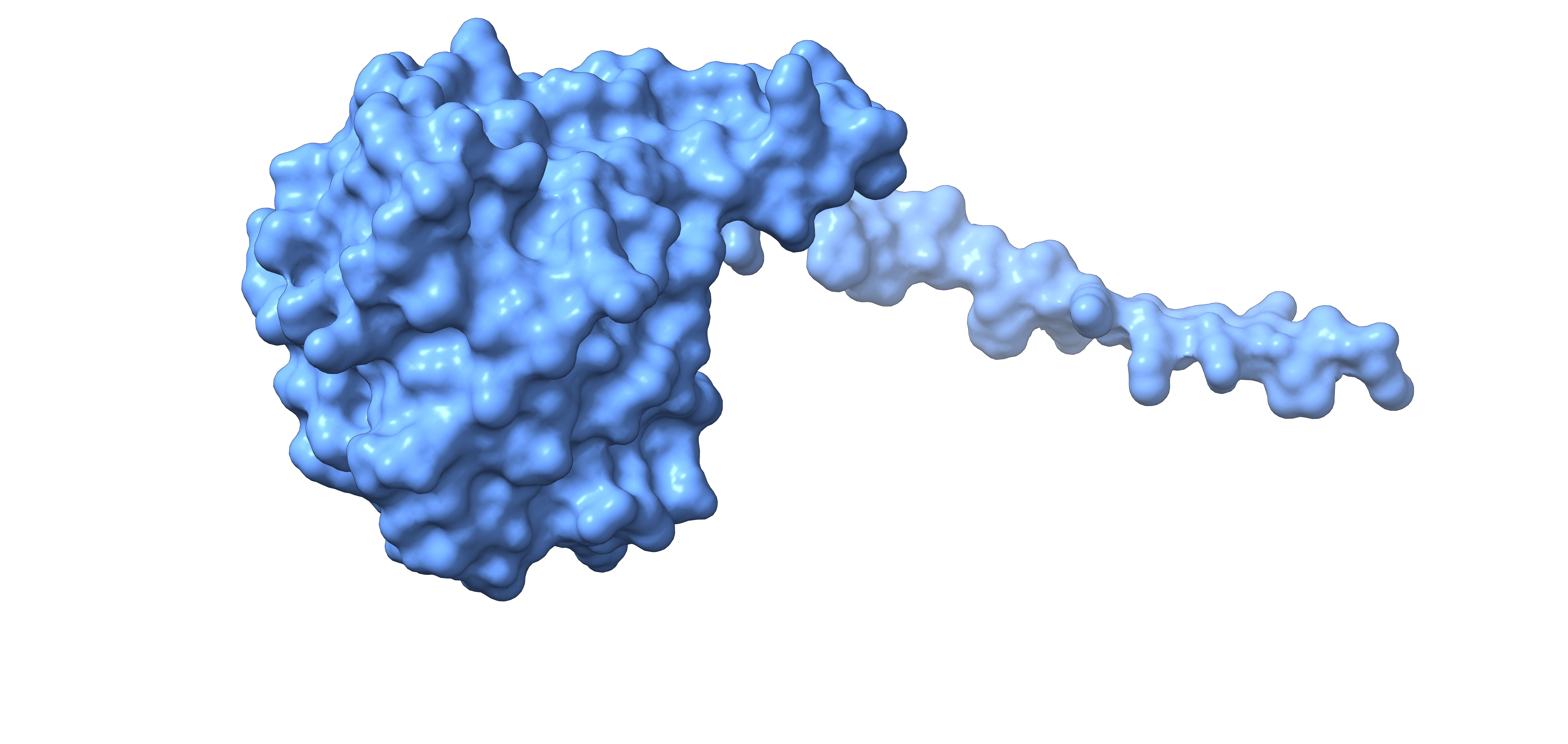

Supplement: S2 File — (ZIP) [file pone.0309738.s002.zip › USMAN PAPER/Structure of Enzymes/SOD-S.jpg]

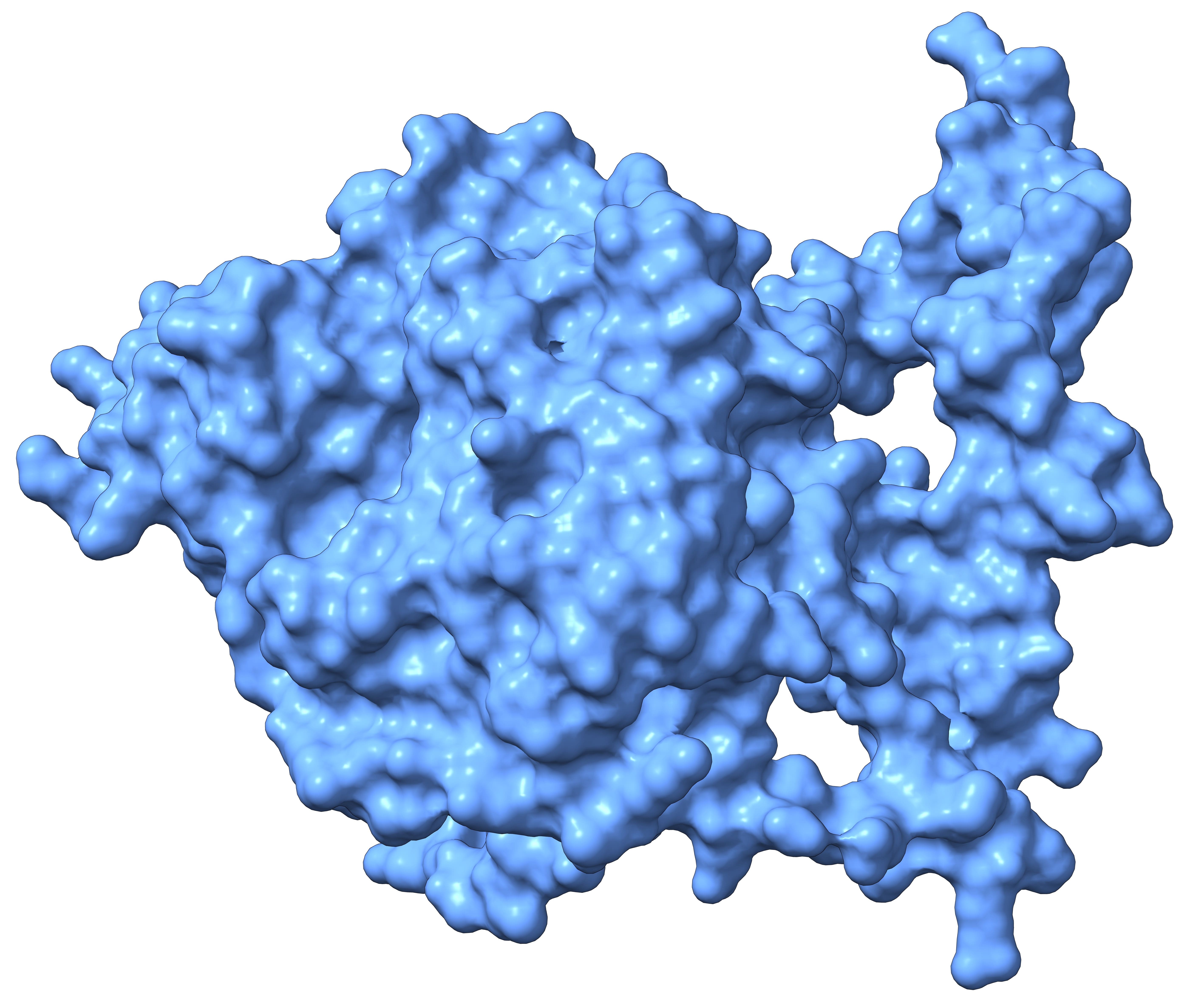

Supplement: S2 File — (ZIP) [file pone.0309738.s002.zip › USMAN PAPER/Structure of Enzymes/CATALASE-Sjpg.jpg]

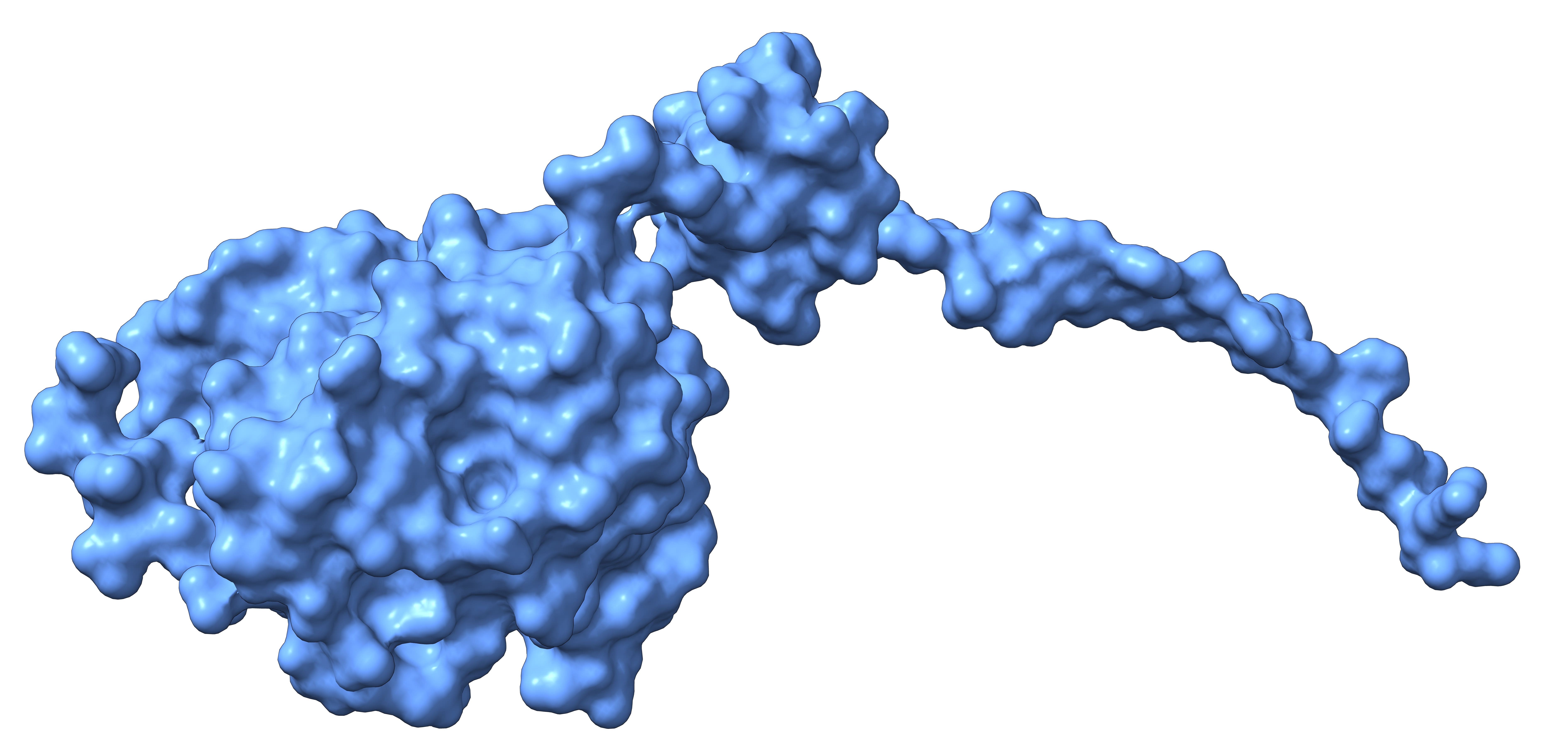

Supplement: S2 File — (ZIP) [file pone.0309738.s002.zip › USMAN PAPER/Structure of Enzymes/CHITINASE-S.jpg]

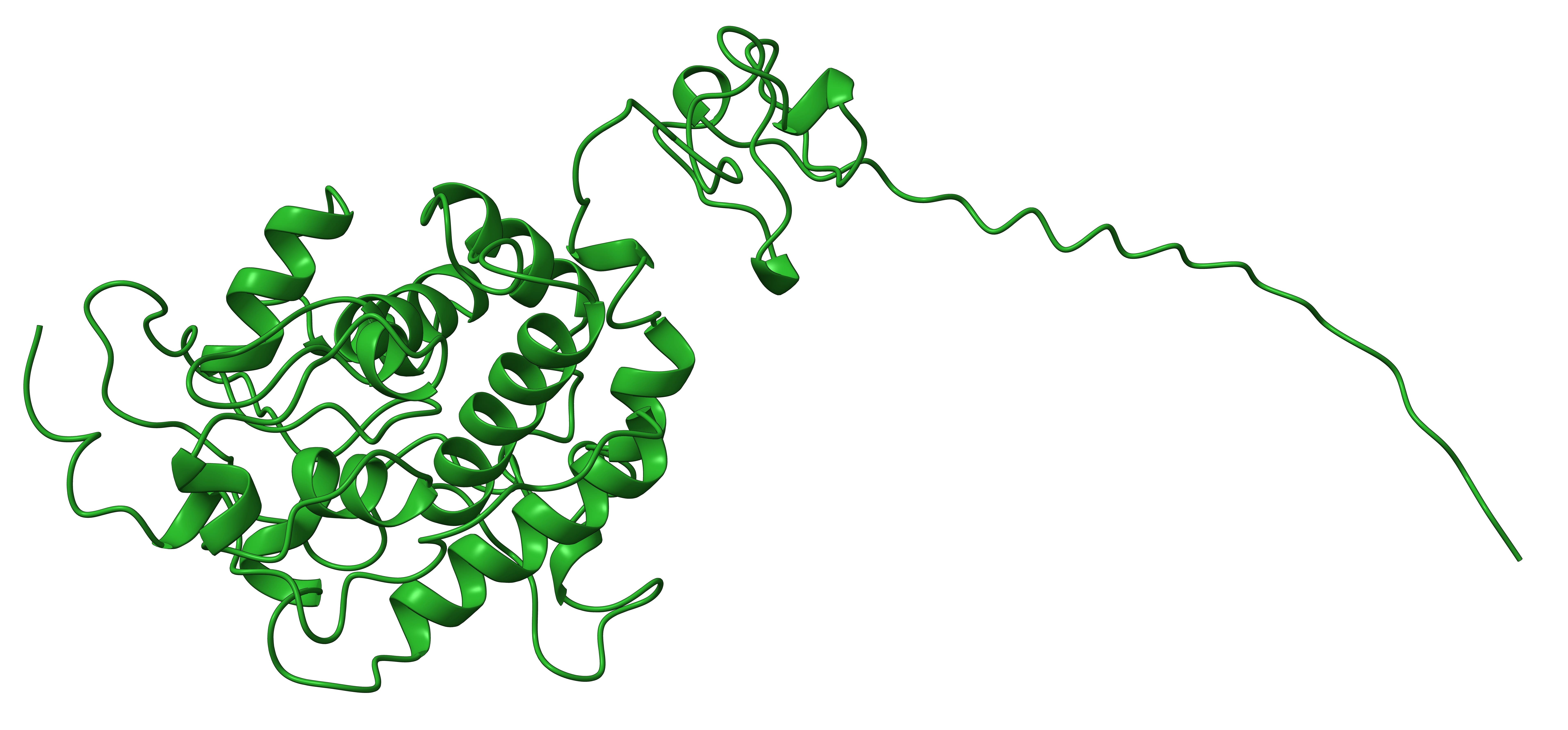

Supplement: S2 File — (ZIP) [file pone.0309738.s002.zip › USMAN PAPER/Structure of Enzymes/CHITINASE-R.jpg]

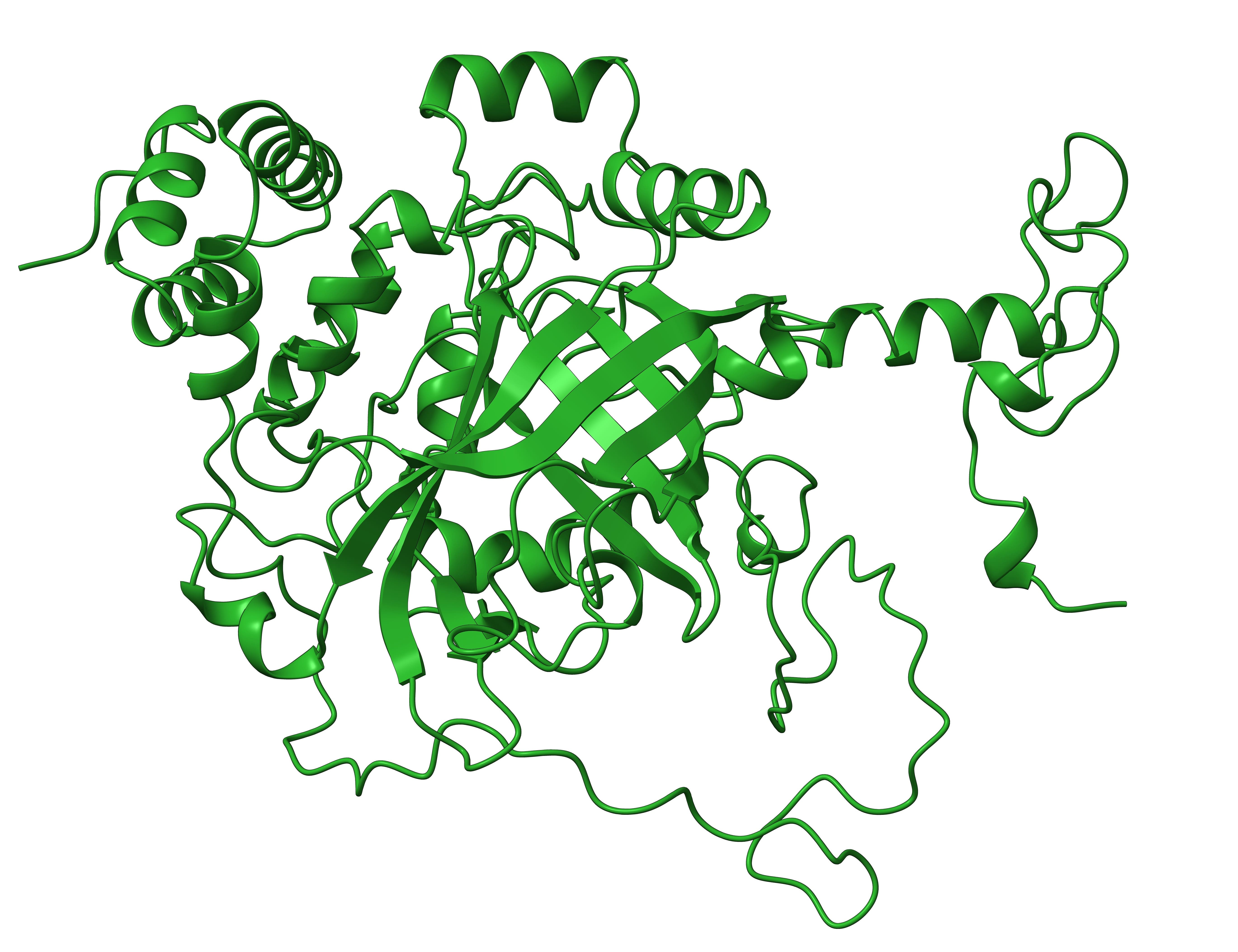

Supplement: S2 File — (ZIP) [file pone.0309738.s002.zip › USMAN PAPER/Structure of Enzymes/CATALASE-R.jpg]

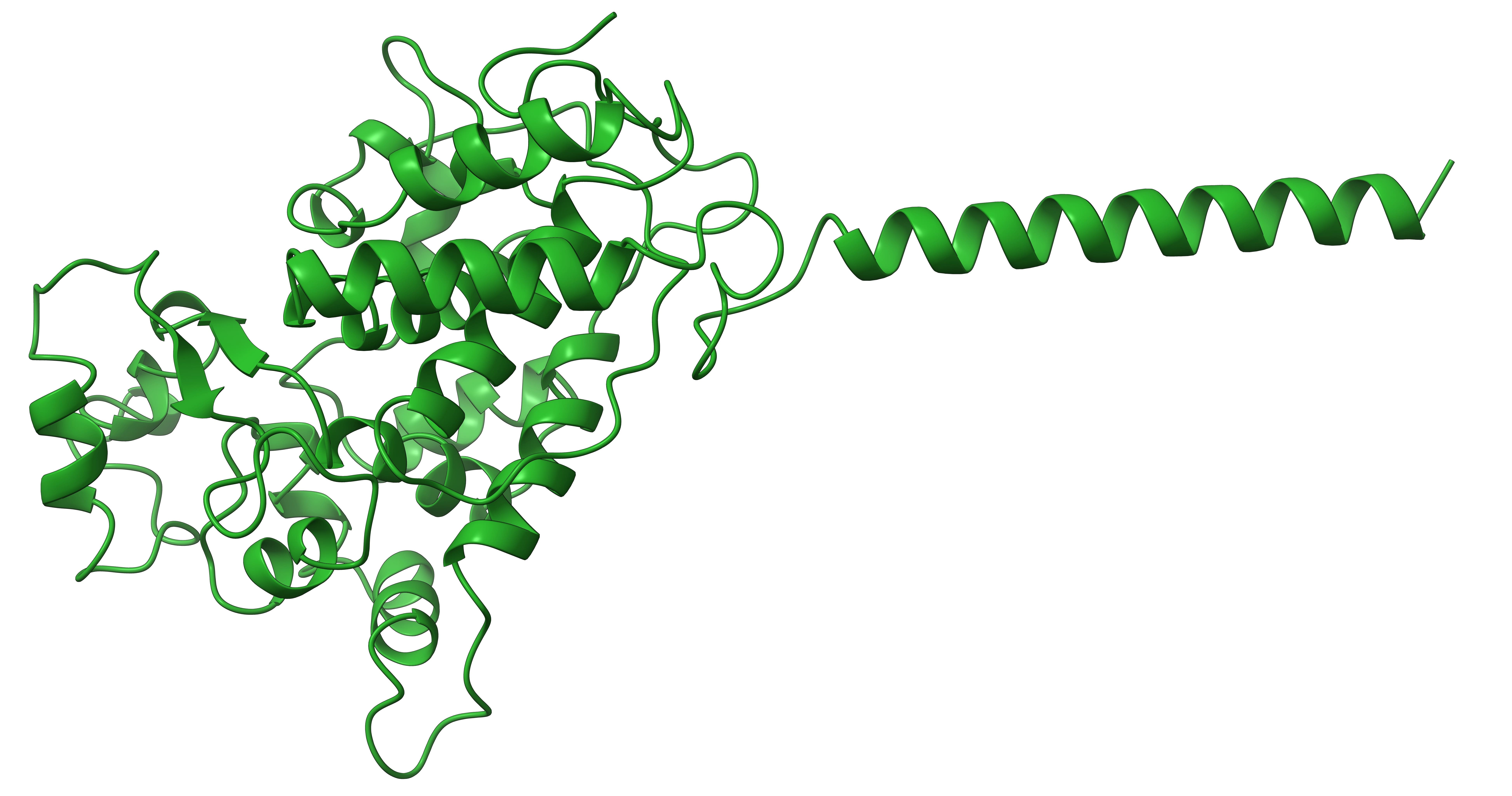

Supplement: S2 File — (ZIP) [file pone.0309738.s002.zip › USMAN PAPER/Structure of Enzymes/PEROXIDASE-R.jpg]

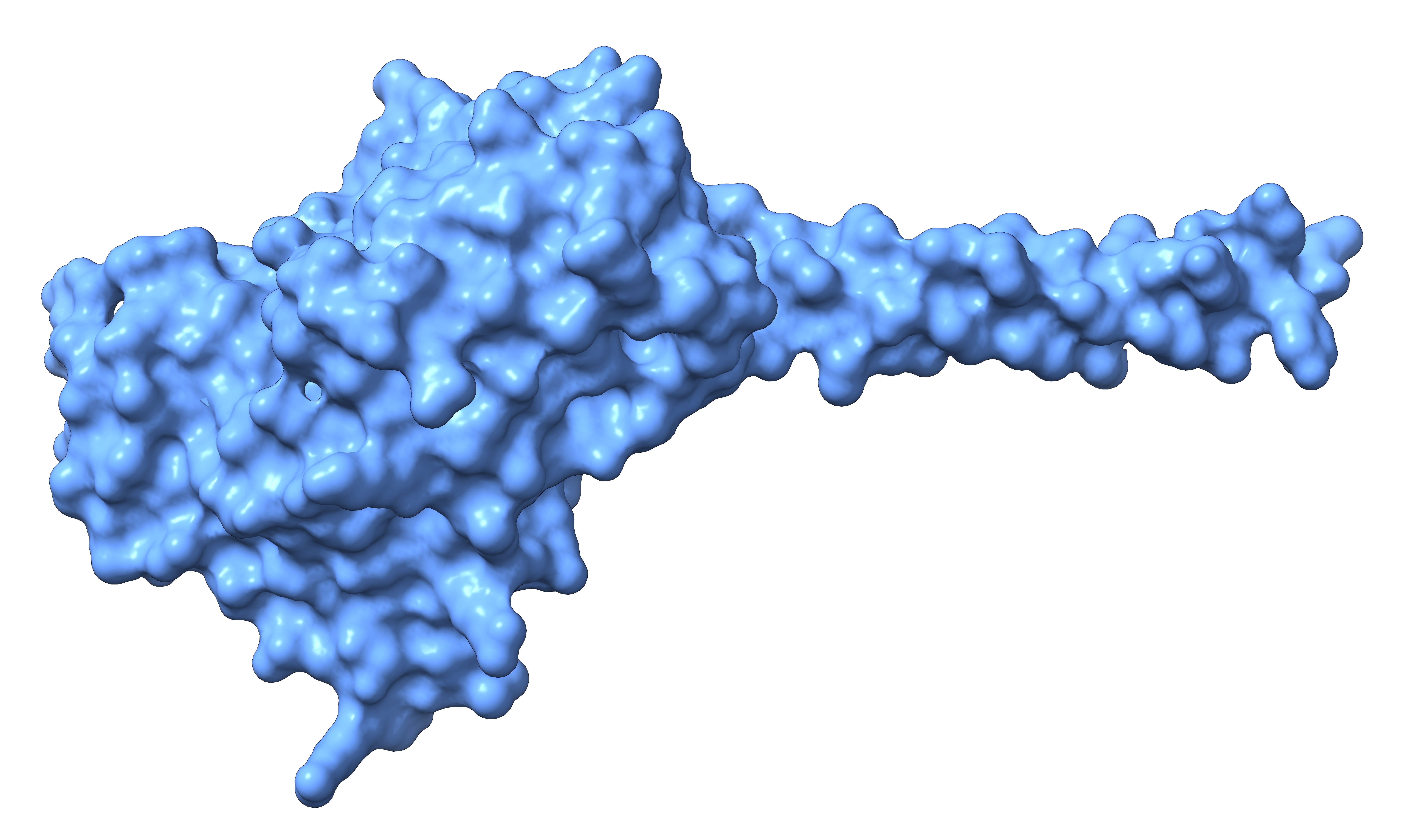

Supplement: S2 File — (ZIP) [file pone.0309738.s002.zip › USMAN PAPER/Structure of Enzymes/PEROXIDASE-S.jpg]

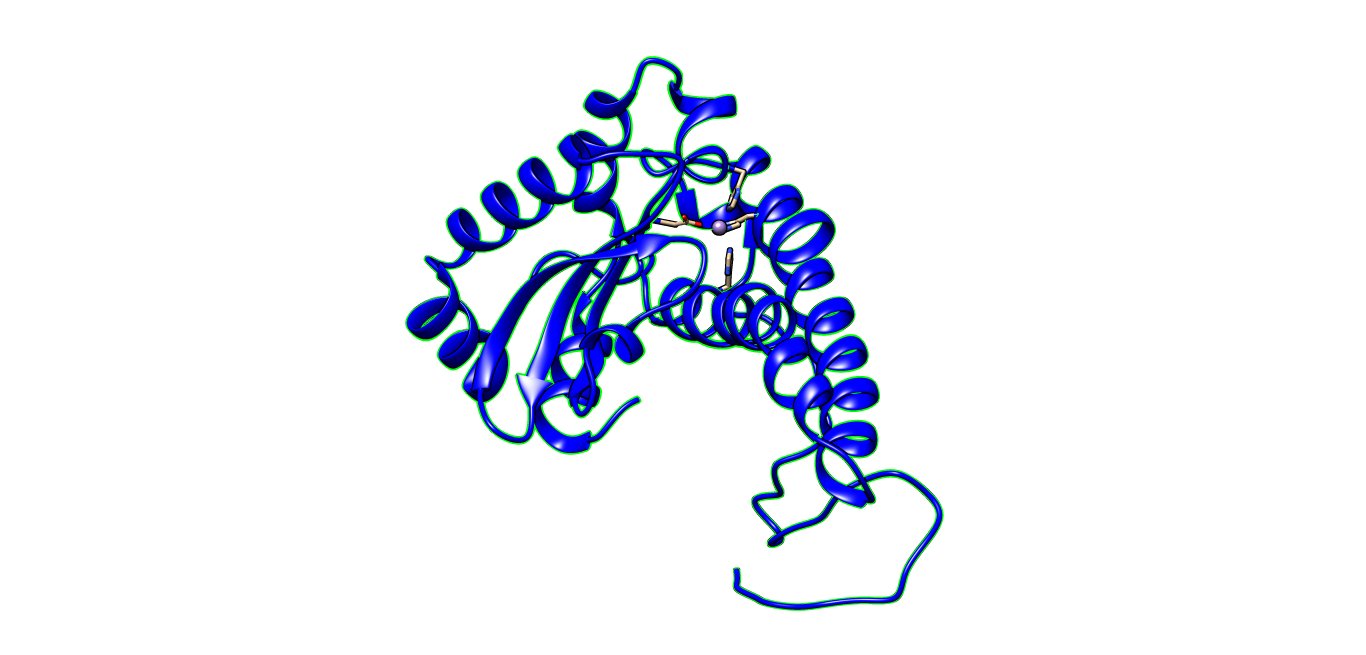

Supplement: S2 File — (ZIP) [file pone.0309738.s002.zip › USMAN PAPER/Enzymes and Metal Ions Data/METAL IONS EFFECTS/SOD/SOD-MN_1.jpg]

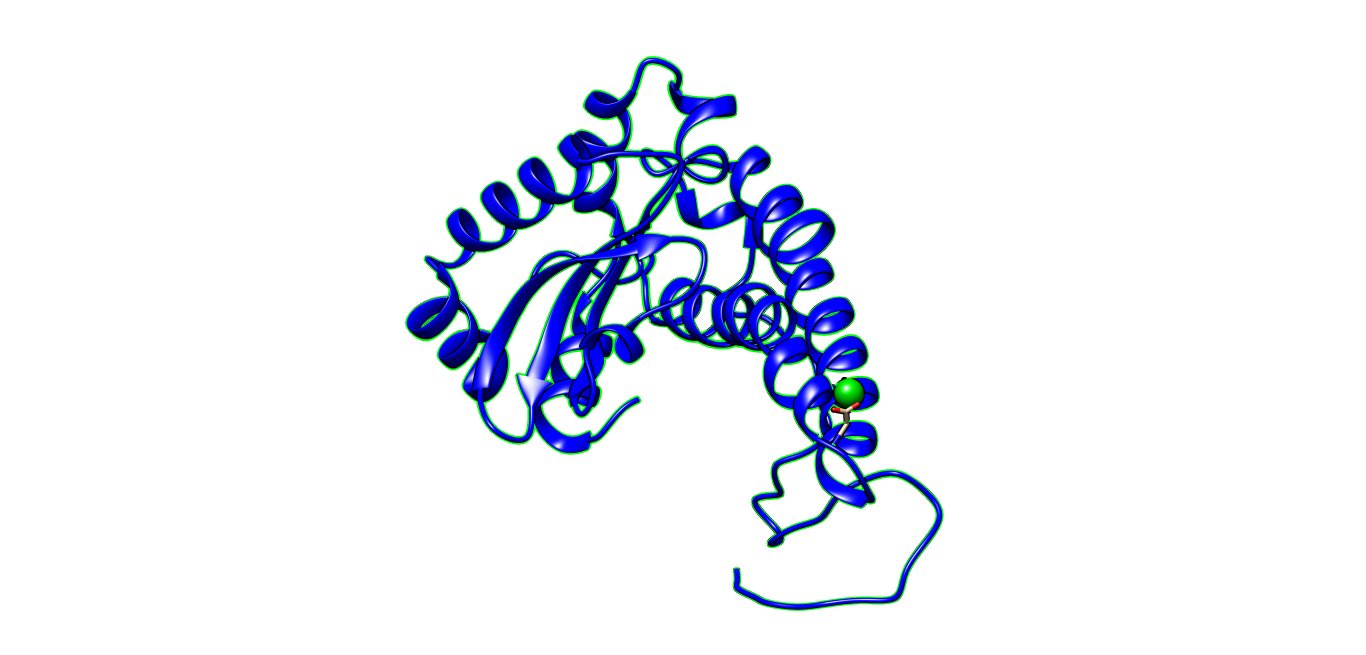

Supplement: S2 File — (ZIP) [file pone.0309738.s002.zip › USMAN PAPER/Enzymes and Metal Ions Data/METAL IONS EFFECTS/SOD/SOD-BA_1.jpg]

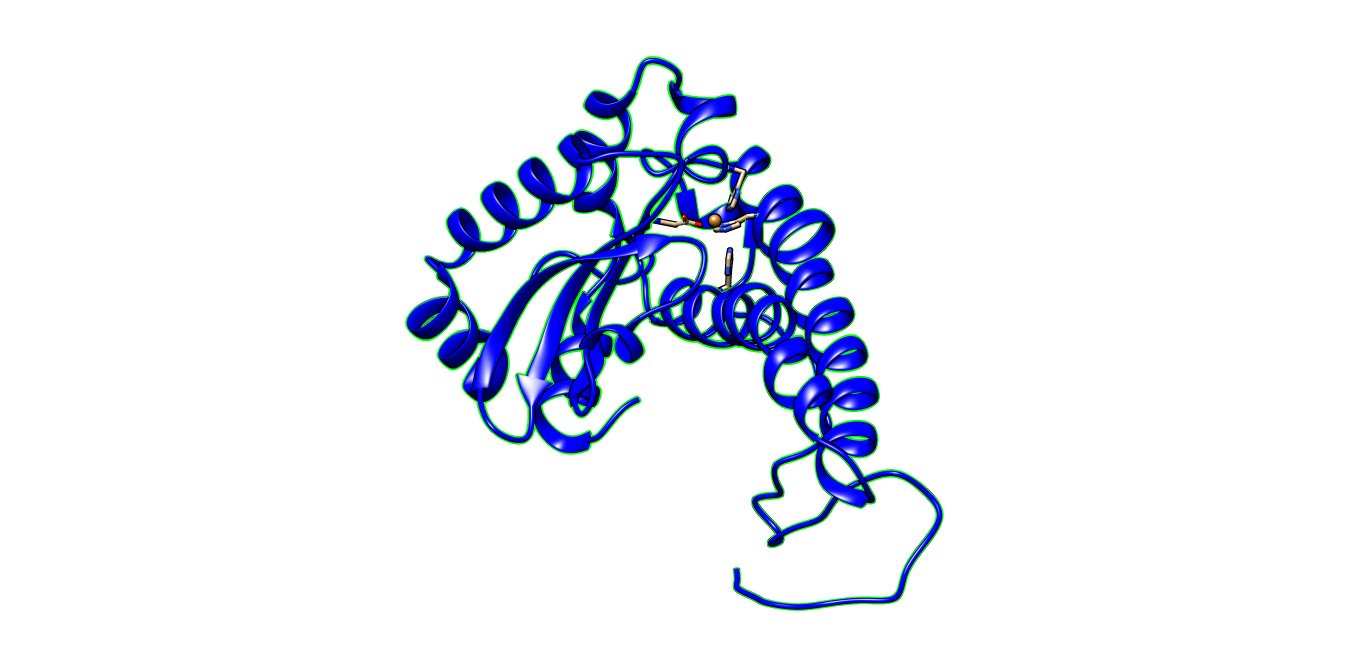

Supplement: S2 File — (ZIP) [file pone.0309738.s002.zip › USMAN PAPER/Enzymes and Metal Ions Data/METAL IONS EFFECTS/SOD/SOD-CU_1.jpg]

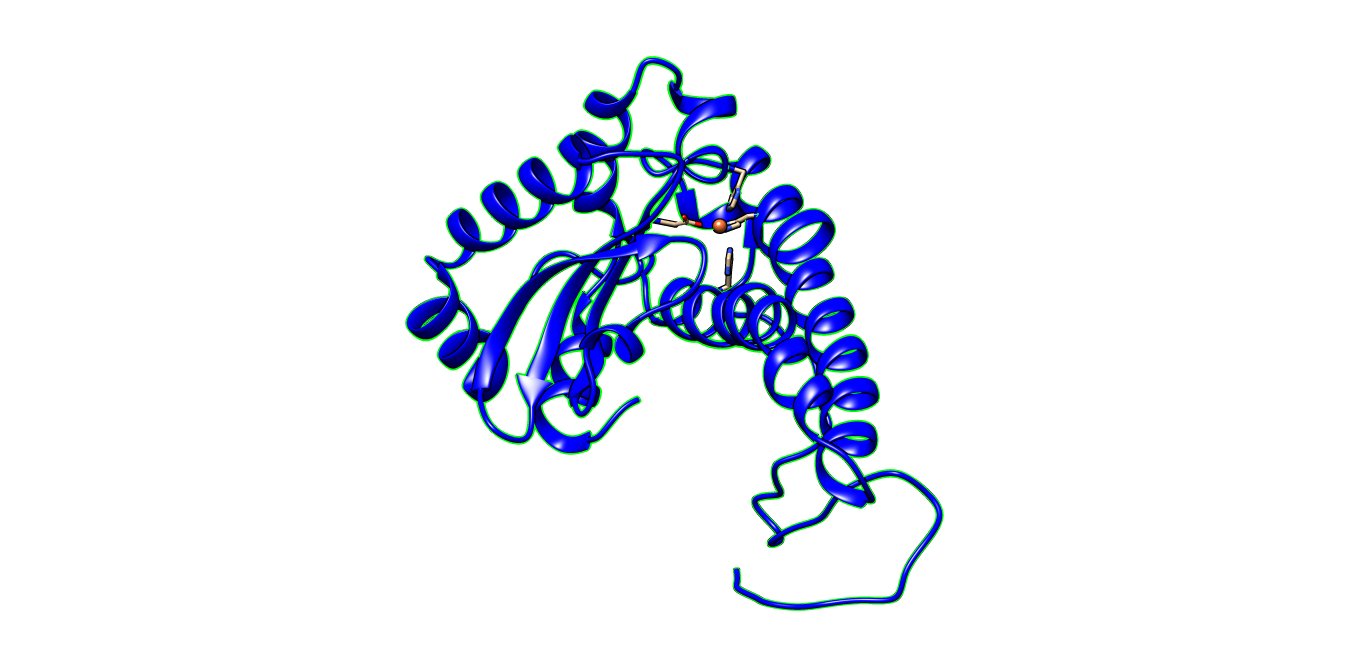

Supplement: S2 File — (ZIP) [file pone.0309738.s002.zip › USMAN PAPER/Enzymes and Metal Ions Data/METAL IONS EFFECTS/SOD/SOD-FE_1.jpg]

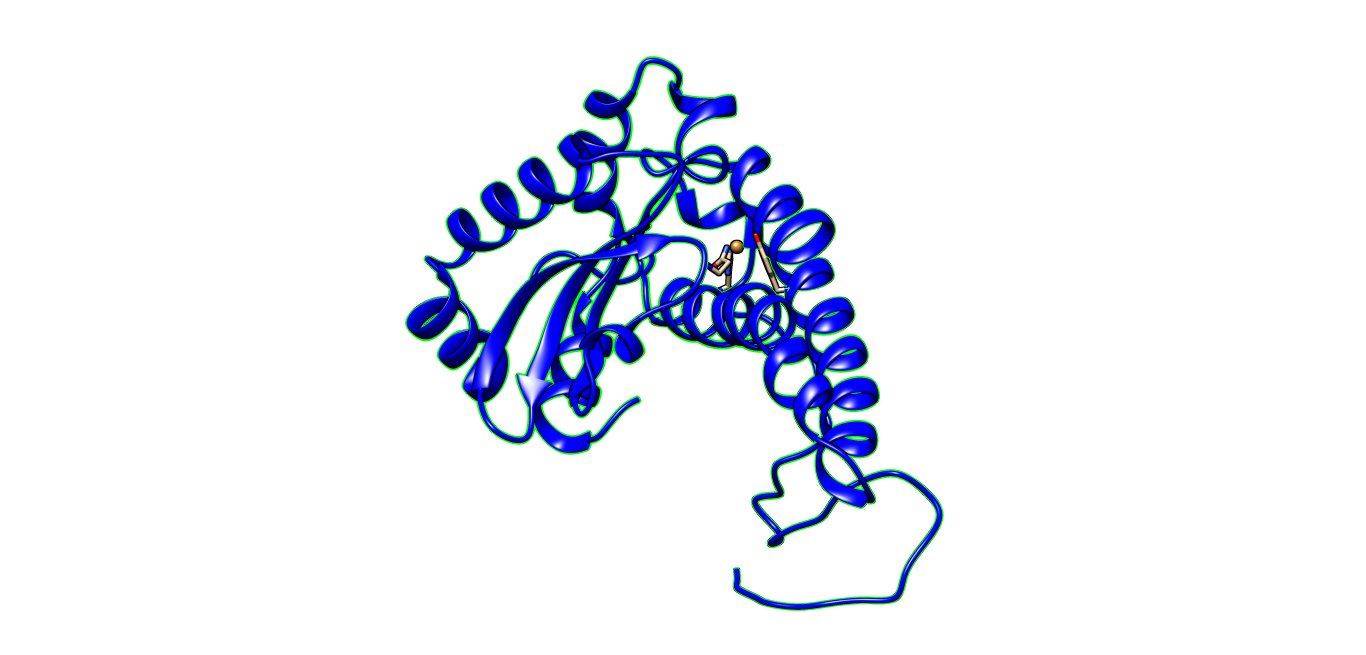

Supplement: S2 File — (ZIP) [file pone.0309738.s002.zip › USMAN PAPER/Enzymes and Metal Ions Data/METAL IONS EFFECTS/SOD/SOD-CU1_1.jpg]

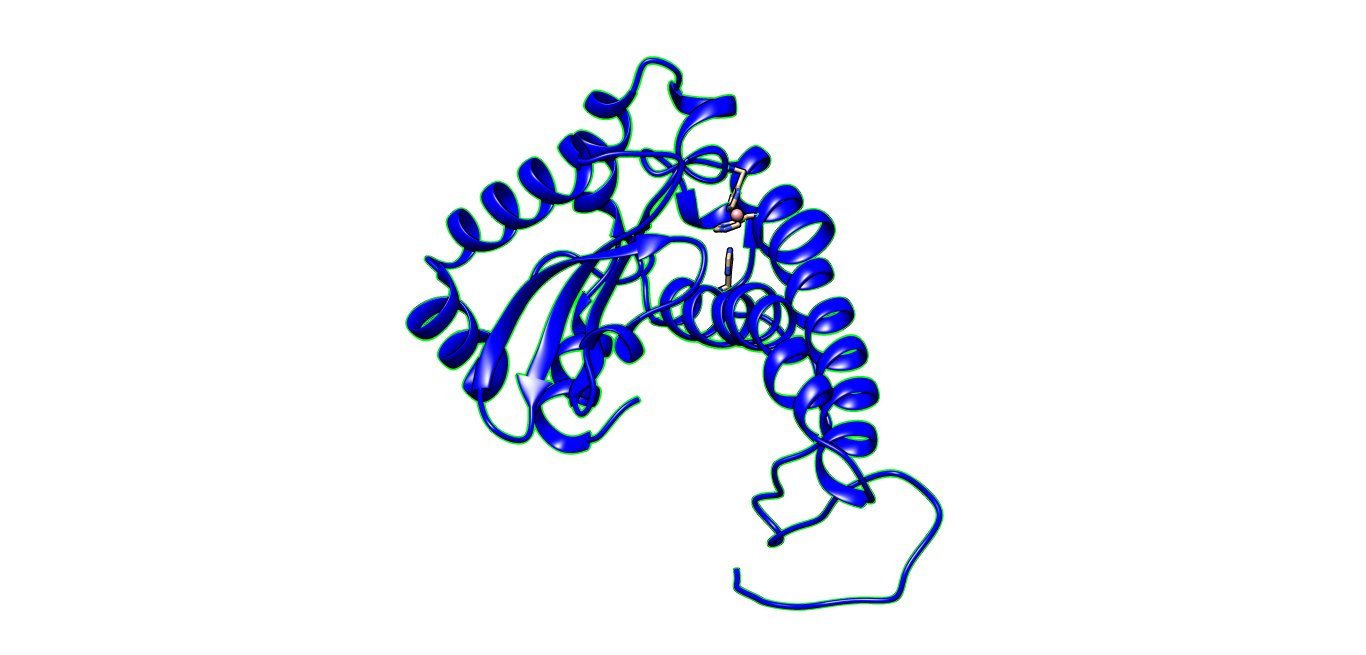

Supplement: S2 File — (ZIP) [file pone.0309738.s002.zip › USMAN PAPER/Enzymes and Metal Ions Data/METAL IONS EFFECTS/SOD/SOD-CO_1.jpg]

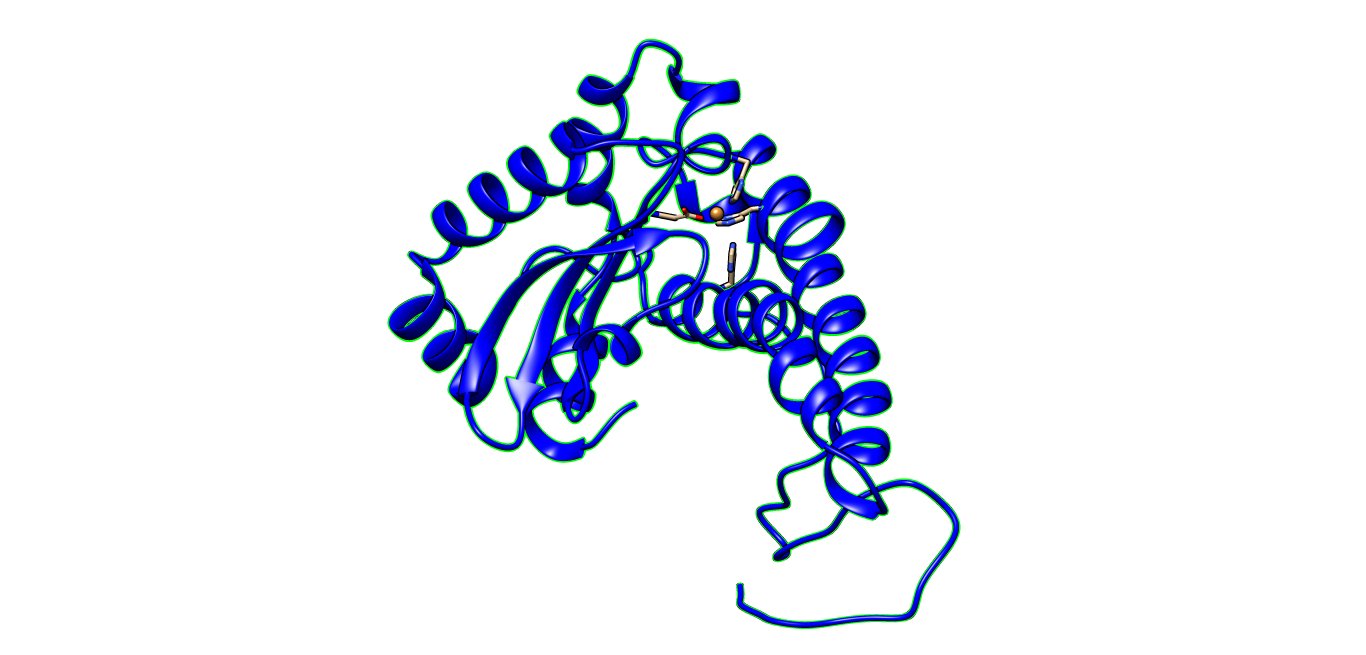

Supplement: S2 File — (ZIP) [file pone.0309738.s002.zip › USMAN PAPER/Enzymes and Metal Ions Data/METAL IONS EFFECTS/SOD/SOD-CA_1.jpg]

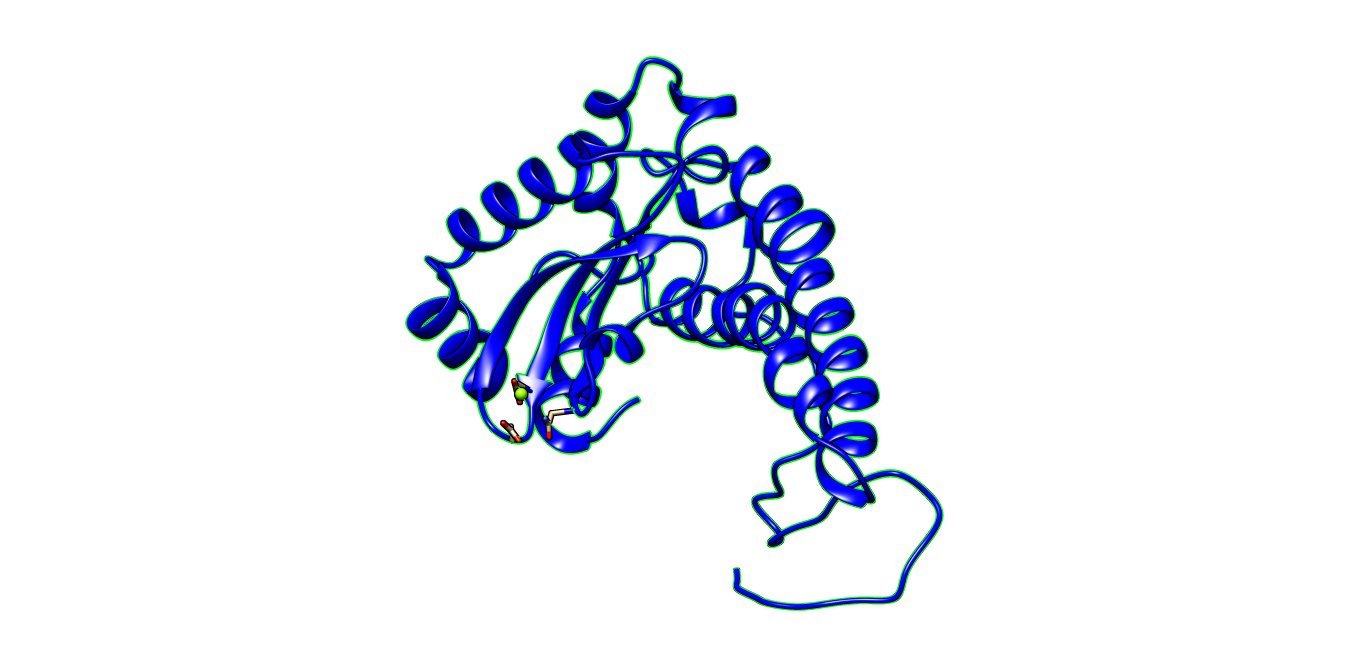

Supplement: S2 File — (ZIP) [file pone.0309738.s002.zip › USMAN PAPER/Enzymes and Metal Ions Data/METAL IONS EFFECTS/SOD/SOD-MG_1.jpg]

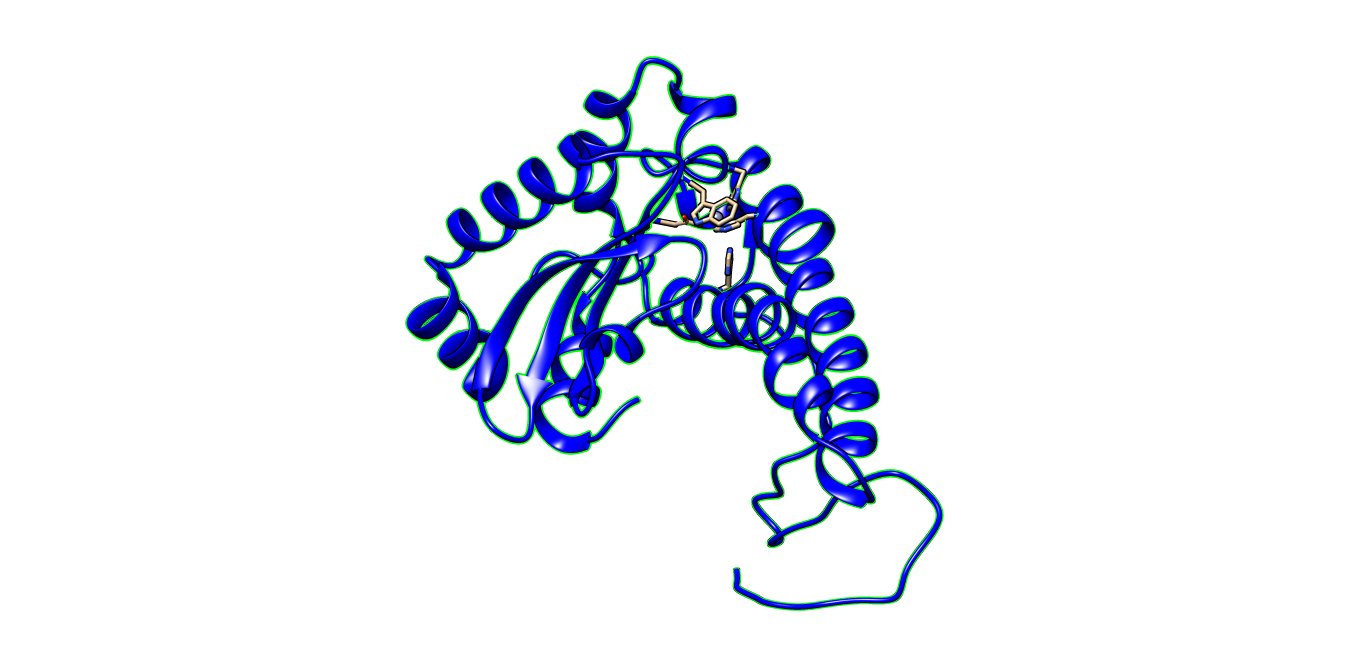

Supplement: S2 File — (ZIP) [file pone.0309738.s002.zip › USMAN PAPER/Enzymes and Metal Ions Data/METAL IONS EFFECTS/SOD/SOD-ZN_1.jpg]

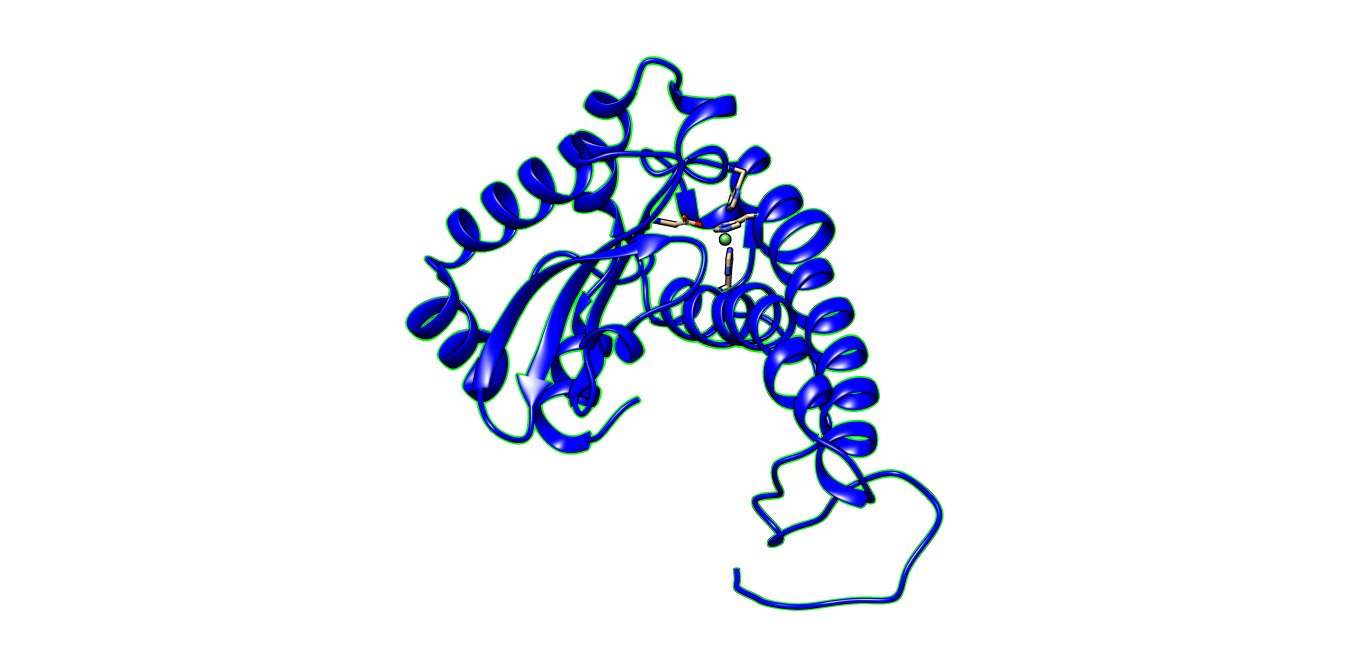

Supplement: S2 File — (ZIP) [file pone.0309738.s002.zip › USMAN PAPER/Enzymes and Metal Ions Data/METAL IONS EFFECTS/SOD/SOD-NI_1.jpg]
